# WormTensor: a clustering method for time-series whole-brain activity data from *C. elegans*

**DOI:** 10.1186/s12859-023-05230-2

**Published:** 2023-06-16

**Authors:** Koki Tsuyuzaki, Kentaro Yamamoto, Yu Toyoshima, Hirofumi Sato, Manami Kanamori, Takayuki Teramoto, Takeshi Ishihara, Yuichi Iino, Itoshi Nikaido

**Affiliations:** 1 Laboratory for Bioinformatics Research RIKEN Center for Biosystems Dynamics Research, Wako, Saitama 351-0198 Japan; 2 Department of Biological Sciences, Graduate School of Science, The University of Tokyo, Bunkyo-ku, Tokyo, 113-0033 Japan; 3 Department of Biology, Faculty of Sciences, Kyushu University, 744, Motooka, Nishi-ku, Fukuoka, 819-0395 Japan; 4 Bioinformatics Course, Master’s/Doctoral Program in Life Science Innovation (T-LSI), School of Integrative and Global Majors (SIGMA), University of Tsukuba, Wako, Saitama 351-0198 Japan; 5 Department of Functional Genome Informatics, Division of Biological Data Science, Medical Research Institute, Tokyo Medical and Dental University (TMDU), Tokyo, 113-8510 Japan

**Keywords:** *C. elegans*, NaCl stimuli, Calcium imaging, Neural activity, Functional modules, Tensor decomposition, Weighting, Consensus clustering

## Abstract

**Background:**

In the field of neuroscience, neural modules and circuits that control biological functions have been found throughout entire neural networks. Correlations in neural activity can be used to identify such neural modules. Recent technological advances enable us to measure whole-brain neural activity with single-cell resolution in several species including $Caenorhabditis\ elegans$. Because current neural activity data in *C. elegans* contain many missing data points, it is necessary to merge results from as many animals as possible to obtain more reliable functional modules.

**Results:**

In this work, we developed a new time-series clustering method, WormTensor, to identify functional modules using whole-brain activity data from *C. elegans*. WormTensor uses a distance measure, modified shape-based distance to account for the lags and the mutual inhibition of cell–cell interactions and applies the tensor decomposition algorithm multi-view clustering based on matrix integration using the higher orthogonal iteration of tensors (HOOI) algorithm (MC-MI-HOOI), which can estimate both the weight to account for the reliability of data from each animal and the clusters that are common across animals.

**Conclusion:**

We applied the method to 24 individual *C. elegans* and successfully found some known functional modules. Compared with a widely used consensus clustering method to aggregate multiple clustering results, WormTensor showed higher silhouette coefficients. Our simulation also showed that WormTensor is robust to contamination from noisy data. WormTensor is freely available as an R/CRAN package https://cran.r-project.org/web/packages/WormTensor.

**Supplementary Information:**

The online version contains supplementary material available at 10.1186/s12859-023-05230-2.

## Background

Nervous systems sense information from the external environment and produce appropriate response behaviors in living animals. Thus, sensory neurons respond to the environmental stimuli, and interneurons and motor neurons are activated in a manner dependent on the activity of the sensory neurons. These neurons showing correlated activities form a functional module, and many efforts have been made to identify such functional modules and understand their dynamics [1, 2].

The nematode $Caenorhabditis\ elegans$ is a model animal in behavioral neuroscience. *C. elegans* are known to migrate toward chemoattractants including sodium chloride (NaCl). This means that changes in NaCl concentration function as external stimuli for the nervous system, and such stimuli can be used to identify functional modules among neurons involved in the induced behavior. In addition, the nervous system of the nematode consists of 302 neurons whose name and connectivity have already been identified anatomically [3]. The small and transparent body of *C. elegans* is suitable for measuring neural activity by calcium imaging [4]. Furthermore, advanced molecular genetics techniques facilitate labeling each neuron in living animals [5–7]. These features enable researchers to obtain functional modules in a comprehensive manner by measuring the whole-brain activity with single-cell resolution.

Several groups, including our own, have already obtained whole-brain activity data from nematodes with neuron identity information [5–10]. However, the obtained whole-brain activity data have several problems that impede the identification of functional modules. In the whole-brain activity data, some neurons are not detected or identified, and are excluded as missing values [6]. The excluded neurons vary among individual animals, and more than half of the neurons are excluded in some experiments. In addition, neural networks show spontaneous and synchronized activities that mask information associated with external stimuli [6]. These activities differ among individual animals, complicating direct comparisons of correlations in neural activities among individual animals. Thus, to find functional modules in whole-brain activity data, we need to resolve the problems of missing values and individual differences in neural states.

Here we present a method WormTensor to find functional modules that are common among individual animals in whole-brain activity data while allowing missing values. We apply the method to our whole-brain data from 24 animals and successfully identify known functional modules. WormTensor uses a distance measure called modified shape-based distance (mSBD) to account for time delay (lag) of cell-cell interactions. In addition, WormTensor uses a tensor decomposition called multi-view clustering based on matrix integration using the HOOI algorithm (MC-MI-HOOI) to detect clusters of cells common to multiple animals and weights for each animal simultaneously. WormTensor is freely available as an R/CRAN package https://cran.r-project.org/web/packages/WormTensor.

## Results

### WormTensor showed high silhouette coefficients

**Fig. 1:**
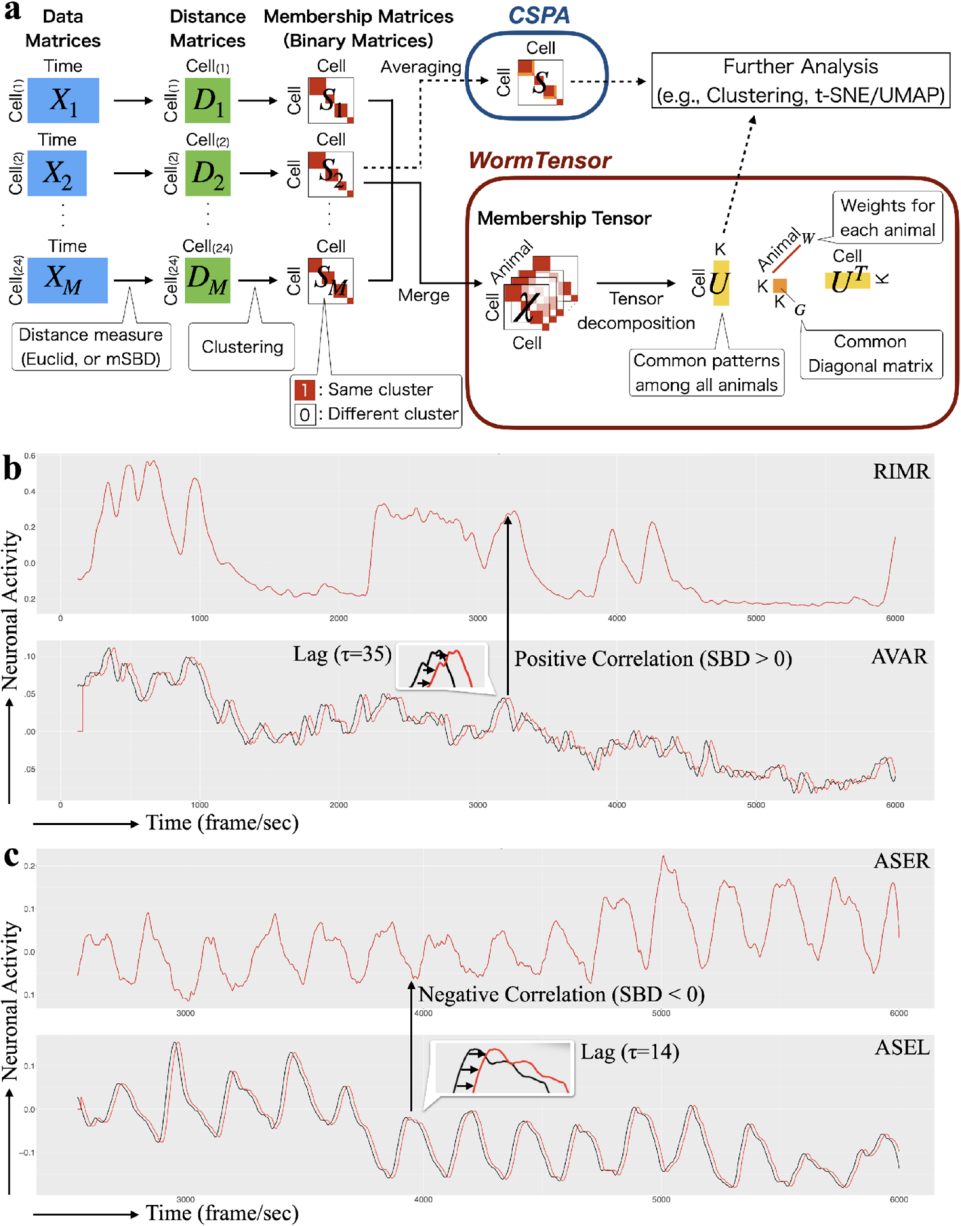
**a** Schematic of WormTensor. The neural activity data matrices measured for *M* animals are transformed into the distance matrices and the membership matrices (binary matrices). In the cluster-based similarity partitioning algorithm (CSPA), the consensus matrix is averaged over all the membership matrices and used for downstream clustering and visualization (t-distributed stochastic neighbor embedding (t-SNE) and uniform manifold approximation and projection (UMAP)). WormTensor, on the other hand, does not take the average, but regards the multiple membership matrices as a third-order tensor, applies tensor decomposition, and uses the computed *K* dimensional factor matrix U for downstream analysis. WormTensor also generates weight vector W, which contains the weights for data from each of the animals. **b** Neuronal activities of neurons with the same phase. In this case, the positive correlation coefficient between AVAR and RIMR is maximized when translating AVAR to the right by $\tau = 35$. **c** Neuronal activities of neurons with reverse phases. In this case, the negative correlation coefficient between ASEL and ASER is minimized when translating ASEL to the right by $\tau = 14$. mSBD handles both (**b**) and (**c**) cases in a unified manner by taking the absolute value of the correlation coefficient

To evaluate the effectivity of usage of mSBD and MC-MI-HOOI, we tested all the combinations of two distance measures (Euclidean distance and mSBD) and two clustering methods (*cluster-based similarity partitioning algorithm* (CSPA) [11, 12] and MC-MI-HOOI) (Fig. 1a). For the details, see the Material and Methods section.

**Fig. 2:**
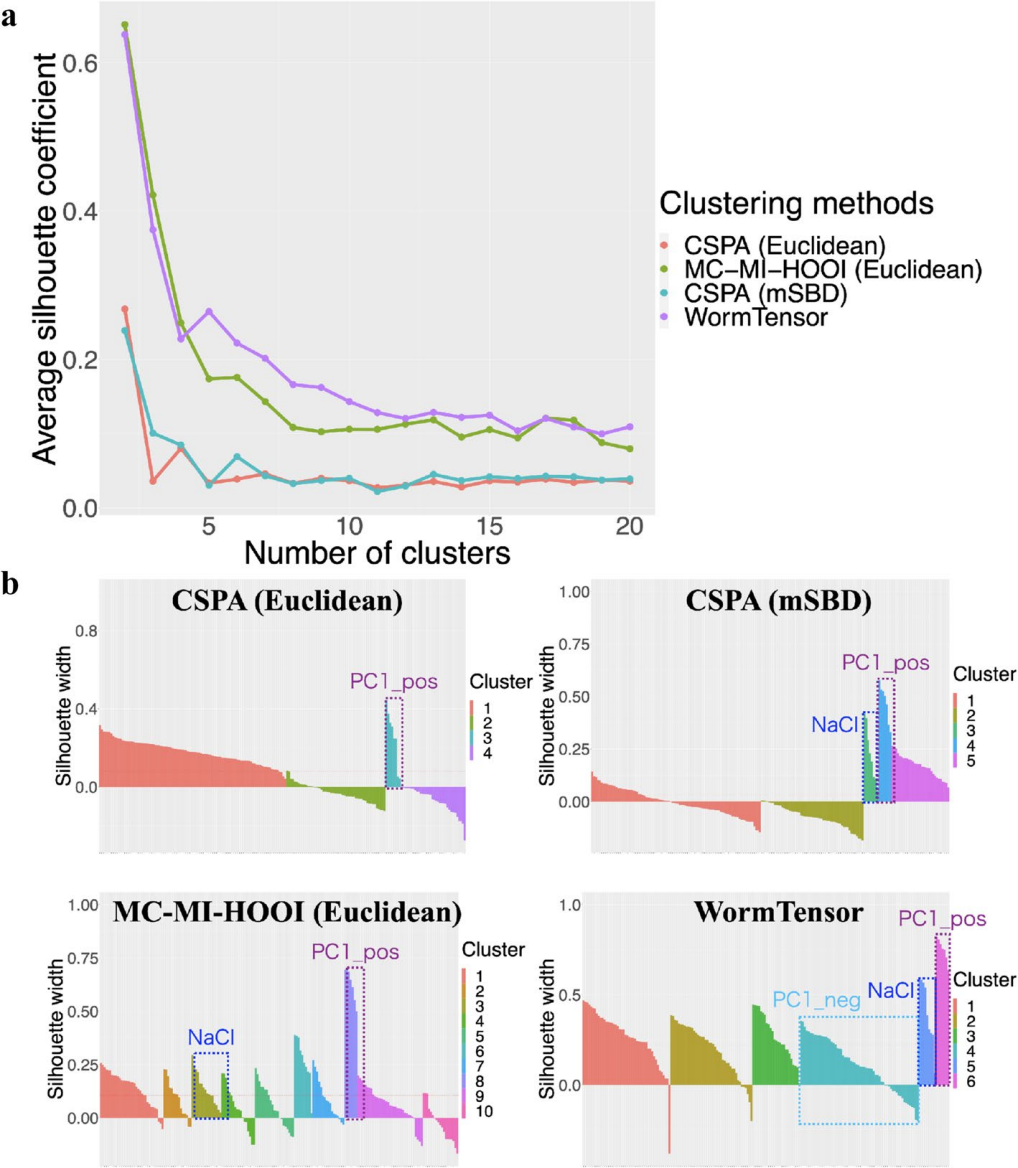
Estimation of the number of clusters. The number of clusters was estimated by silhouette coefficients and prior knowledge about the movement of *C. elegans*. The cluster-based similarity partitioning algorithm (CSPA) and WormTensor with Euclidean distance and modified shape-based distance (mSBD) are performed with the number of clusters (2–20). **a** The *x*-axis represents the number of clusters, and the *y*-axis represents the average silhouette coefficient for all the cells for each number of clusters. **b** The silhouette coefficient for each cell in the optimal number of clusters

For all the combinations, we quantitatively evaluated the clustering results using silhouette coefficients [13] (see Material and Methods). The values of silhouette coefficients were calculated in each cell of each animal to show how each cluster is aggregated compared to the other clusters. The averaged value of the silhouette coefficients of all the cells was calculated in each number of clusters (Fig. 2a). The values showed that, regardless of the number of clusters and the distance measures, MC-MI-HOOI was able to capture more aggregated clusters compared with CSPA. Additionally, mSBD further outperformed Euclidean distance, which suggests that there are lags of cell–cell interactions between neurons in *C. elegans*, and correcting for the shift-invariance contributes to detecting clusters that are repeated across animals.

### Estimation of the optimal number of clusters

Using the cellular labels listing the known functional modules (Additional files 1 and 2), we found that the NaCl stimulus-related cells and principal component 1-related cells with positive coefficients (PC1_pos-related) cells were relatively easy to detect as clusters in many clustering methods. PC1_pos-related cells were heavily weighted in the first principal component of whole-brain activity data in a previous study [8] and are involved in forward and backward locomotion of *C. elegans*.

NaCl- and PC1_pos-related cells could be differentiated without negative silhouette coefficients when WormTensor considered six clusters (Fig. 2b). Therefore, we regarded this value as the optimal number of clusters for WormTensor and used six clusters in further analysis. For MC-MI-HOOI with Euclidean distance and CSPA with mSBD, similar results were obtained when the number of clusters was 10 and 5, respectively. For CSPA with Euclidean distance, however, only PC1_pos-related cells were detected as a cluster. For the details of these clustering results, see Additional files 2, 3, 4, 5, 6, 7 and 8).

### WormTensor detected NaCl stimulus-related and movement-related cells

**Fig. 3:**
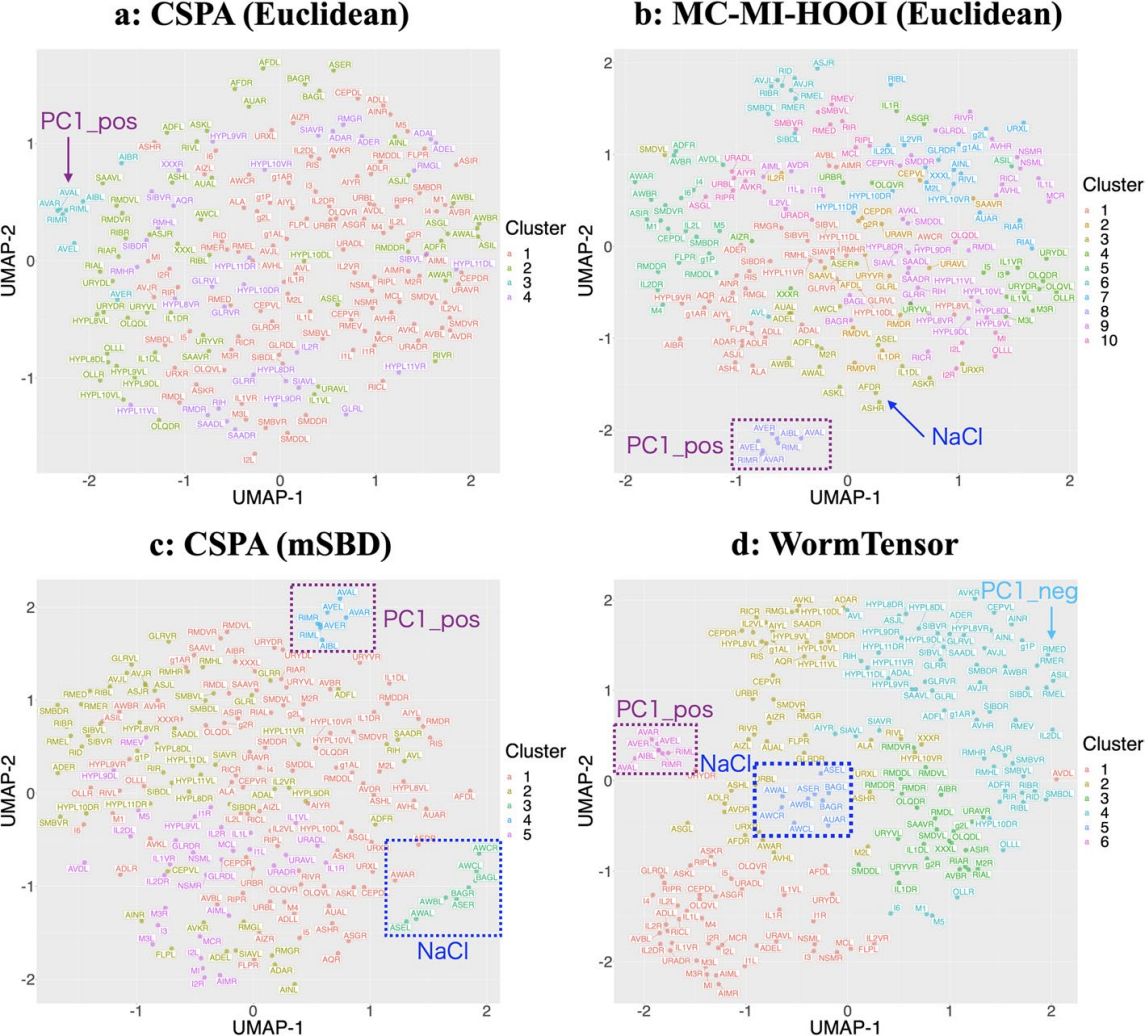
Results of clustering with the optimal number of clusters utilizing t-distributed stochastic neighbor embedding (t-SNE) and uniform manifold approximation and projection (UMAP). **a** The clustering results of the cluster-based similarity partitioning algorithm (CSPA) with Euclidean distance. **b** The clustering results of multi-view clustering based on matrix integration using the HOOI algorithm (MC-MI-HOOI) with Euclidean distance. **c** The clustering results of CSPA with modified shape-based distance (mSBD). **d** The clustering results of WormTensor, utilizing MC-MI-HOOI with mSBD

Figure 3 shows the UMAP plots for all the combinations of the distance measures and the clustering methods. As the plots of WormTensor (Fig. 3d) and CSPA with mSBD (Fig. 3c) show, NaCl stimulus-related cells (cluster $\#5$ for WormTensor, $\#3$ for CSPA with mSBD) and PC1_pos-related cells (cluster $\#6$ for WormTensor, $\#4$ for CSPA with mSBD) were separated as distinct clusters.

**Fig. 4:**
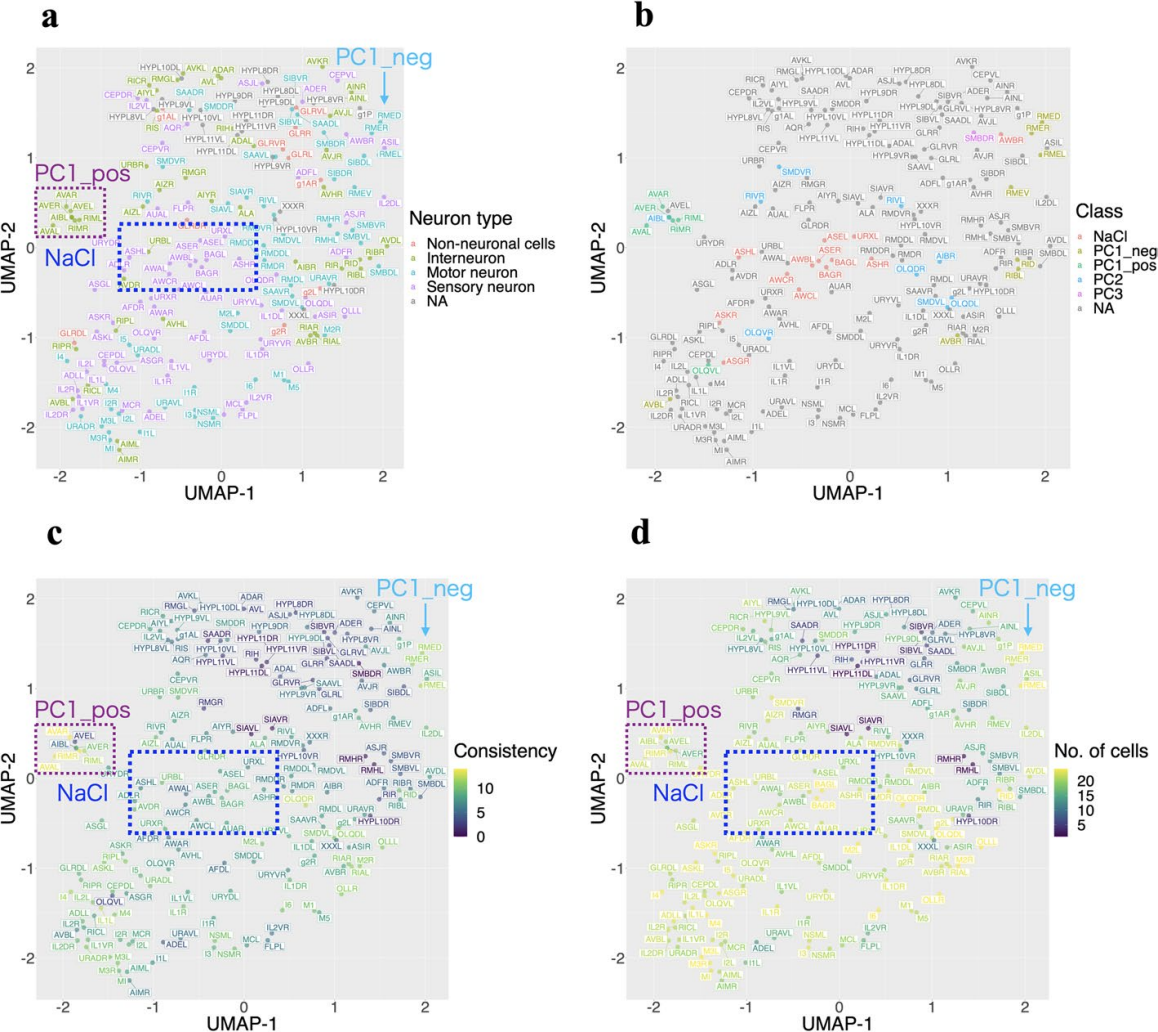
Results of WormTensor utilizing t-distributed stochastic neighbor embedding (t-SNE) and uniform manifold approximation and projection (UMAP). **a** Neuron type (based on WormWiring annotation). **b** The labels of movement in *C. elegans*. **c** Consistency between the results of hierarchical clustering in each animal and WormTensor. **d** The number of identified cells

Using prior knowledge about the neuronal cells (Additional files 1 and 2), we interpreted some clusters (Fig. 4). For example, the cells that formed clusters are not clustered according to the neuron type, e.g., “Non-neuronal cells”, “Interneuron”, “Motor neuron”, and “Sensory neuron”, based on WormWiring annotation (https://wormwiring.org) (Fig. 4a). Rather, it showed a structure that followed the known functional modules, such as NaCl stimulus (cluster $\#5$), PC1_pos (cluster $\#6$), and principal component 1-related cells with negative coefficients (cluster $\#4$, PC1_neg [8]) (Fig. 4b). We assessed “consistency” between the clusters of WormTensor calculated from all the animals and the clusters calculated in each animal (see Materials and Methods). By using this measure, these three clusters were found to be reproducibly detected in the majority of animals (Fig. 4c). For another presentation of the results of Figs. 3 and 4 with the t-SNE coordinates, see Additional files 9 and 10.

In addition to the three clusters above, another cluster was also annotated based on previous reports. We also evaluated whether a set of annotation terms is enriched within each cluster using the hypergeometric test and Benjamini-Hochberg method [14] to correct for the multiple testing problem (Additional file 11).

According to this approach, for example, some PC2-related cells (these cells were heavily weighted in the second principal component of whole-brain activity data in the previous study [8] that are involved in turning movement of *C. elegans*) were enriched in cluster $\#3$ (Table 1).

**Table 1:** Summary of the clustering results of WormTensor

Cluster No.	Our annotations	Example cells
#1	Unknown	–
#2	PC2-related cells	SMDVR, RIVL/R
#3	PC2-related cells	AIBR, OLQDL/R, SMDVL
#4	PC1_neg-related cells and some epidermal cells (The number of identified cells is relatively low; see Fig. 4d)	RIBL, RMED, RMEL/R, RMEV
#5	NaCl stimulus-related cells	ASEL/R, AWBL, AWCL/R, BAGL/R
#6	PC1_pos-related cells	AVAL/R, AVER, RIML/R, (AIBL)

**Table 2:** Summary of the *C. elegans* animals used

Animal no.	No. of cells	Frame/sec	QC by visual inspection
#1	161	4.12	PASS
#2	182	5.72	PASS
#3	158	5.71	WARNING (temporary abnormal waveform)
#4	180	5.71	PASS
#5	197	5.71	PASS
#6	180	5.71	PASS
#7	196	5.72	PASS
#8	196	5.72	WARNING (temporary abnormal waveform)
#9	173	5.72	PASS
#10	191	5.71	PASS
#11	157	5.71	PASS
#12	168	5.71	PASS
#13	211	4.12	PASS
#14	231	3.73	PASS
#15	201	3.69	PASS
#16	198	3.69	PASS
#17	202	4.10	PASS
#18	214	4.05	PASS
#19	215	4.05	PASS
#20	203	4.04	FAILURE (severe abnormal waveform)
#21	216	4.04	PASS
#22	181	4.09	PASS
#23	207	4.08	PASS
#24	207	4.07	PASS
#25	156	4.04	WARNING (temporary abnormal waveform)
#26	196	4.06	PASS
#27	220	4.05	PASS
#28	207	3.99	PASS

All neural activity values were measured for 6000 time frames

While AIBL is a PC2-related cell, it is categorized in PC1_pos cluster. This is probably because AIBL is involved in PC2 as well as PC1_pos, as previously shown [8, 15]. Likewise, AVBR, which is one of the PC1_neg-related cells categorized in PC2 cluster, might be involved in both PC1_neg and PC2, as some studies have suggested [8, 16].

Although the NaCl stimulus and PC1_pos-related cells were detected by both WormTensor and CSPA with mSBD, WormTensor was more able to enrich a cluster with PC1_neg-related cells (Fig. 2b and Additional file 11). In consideration of these results together with its high silhouette coefficient (Fig. 2a), WormTensor was found to be the most suitable for capturing functional modules in *C. elegans* compared with the other methods tested in this study.

### WormTensor automatically assigned small weights to animals with a small number of identified cells and noisy data

Unlike CSPA, which assumes that all animals have common clusters, WormTensor is a model that allows differences among animals; in the optimization process, animals without common clusters are automatically evaluated with smaller weights. We further interpreted the weights of WormTensor. We investigated whether the datasets contain any covariates that are correlated with the weights and found at least three possible covariates as follows.

**Fig. 5:**
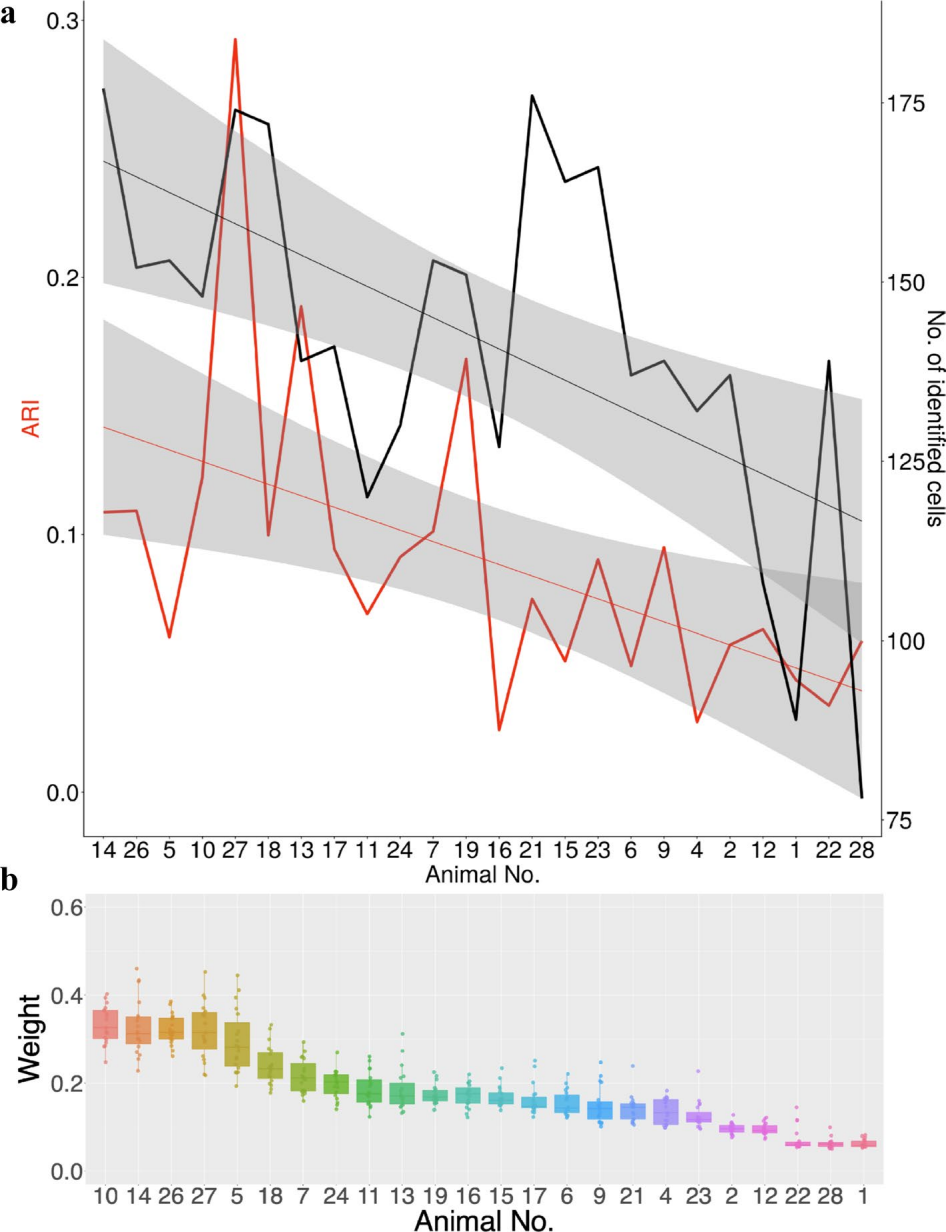
Interpretation of the weights of WormTensor. **a** The *x*-axis represents the animals sorted by their weights in WormTensor with the number of clusters set to 6. the left *y*-axis represents the adjusted Rand index (ARI) value between the result of clustering by WormTensor and Ward’s hierarchical clustering, and the right *y*-axis represents the number of identified cells. **b** The *y*-axis shows the weights of all the animals for different numbers of cluster (2–20) and the *x*-axis shows the animals sorted by their average weights with different numbers of clusters

The first potential covariate is the total number of annotated cells in each animal (Fig. 5a, black line). To evaluate whether this possible covariate is correlated with the weights in the result of WormTensor with 6 clusters, we performed the Cochran–Armitage trend test, and the *p*-value was $5.60E-34$.

The second potential covariate is the similarity between the clustering results of WormTensor and the clustering results of individual animals (Fig. 5a, red line). The similarity was quantified using the adjusted Rand index (ARI [17]) between them, and the *p*-value of the Jonckheere–Terpstra trend test was $9.66E-4$.

Both of these tests were highly significant at the $p < 0.05$ level and thus indicate decreasing trends in the number of identified cells and the similarity of clustering results according to the weights (Fig. 5a). We also investigated the results with different numbers of clusters (2–20) (Fig. 5b) and found that the animals with small weights were generally assigned robustly small values independent of the number of clusters.

**Fig. 6:**
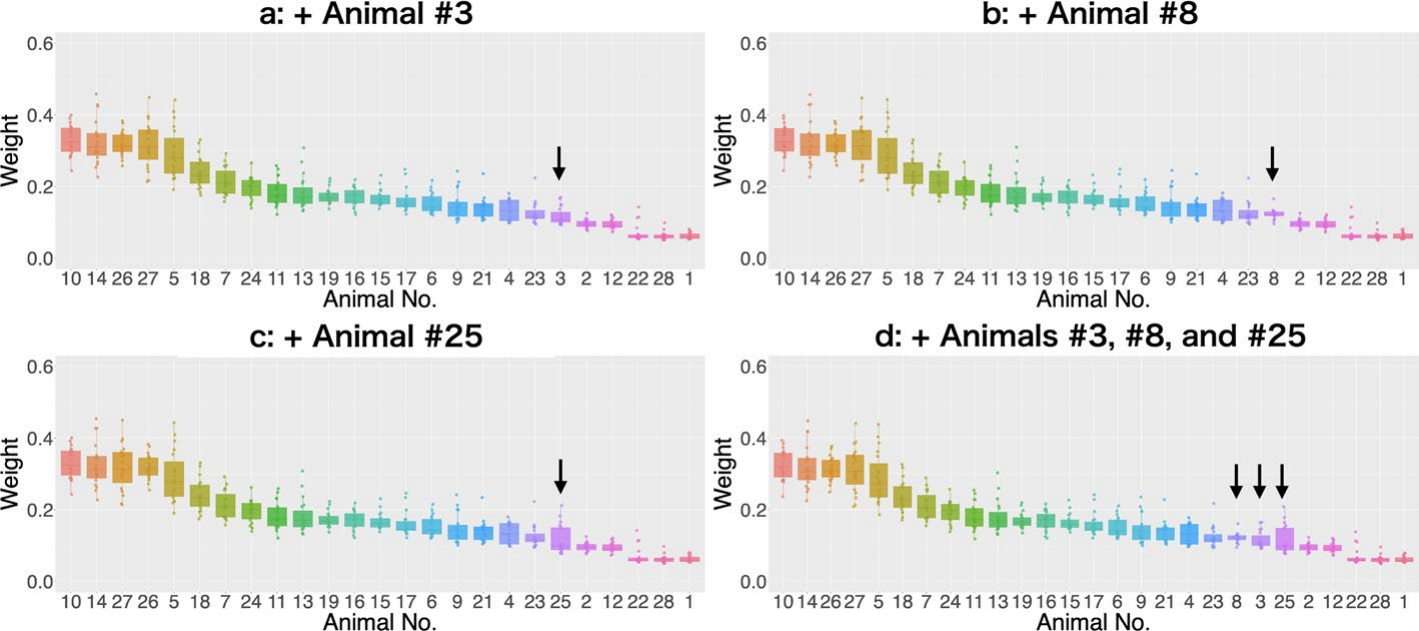
Robustness to noisy data addition. **a**–**c** The order of WormTensor weights of the animals when an additional noisy data matrix was added individually. **d** The order of WormTensor weights of the animals when three noisy data matrices were added at once. The arrows indicate the added noisy datasets

The third potential covariate is the noisiness of temporal patterns in data from each animal. In this work, the data matrices from three animals ($\#3$, $\#8$, and $\#25$) that contained abnormal waveforms in all the cells regardless of cell type and were excluded from the analysis by prior quality control (QC) (Table 2) were added to the WormTensor input individually. The weights for animals $\#3$, $\#8$, and $\#25$ were automatically reduced (Fig. 6a–c), and similar results were obtained when all three animals were input into WormTensor at once (Fig. 6d).

We also added the data matrix from animal $\#20$, whose temporal patterns were contaminated with extremely strong noise. For this animal, however, WormTensor’s effect of reducing weight for noisy data was modest (as it was ranked 8-th according to weight among all the animals, Additional file 12). Further examination revealed that animal $\#20$ had left the holding position of the microfluidic chip during imaging and moved outside of the field of view, which caused an abnormal fluorescence intensity change in all cells (Additional file 12). There are at least two possible explanations for the high weight despite the anomaly of the waveforms. First, the number of cells that could be tracked before the abnormal waves occurred was relatively large, so WormTensor estimated the weights to be high. Second, an outlier distance matrix may have been generated from animal $\#20$ and optimization subsequently overfitted data from the animal.

Although the above covariates alone do not perfectly explain the weights, as all of the above covariates are related to data quality, the property of WormTensor that automatically reduces the weights for such unreliable animals’ datasets is expected to be useful from a QC perspective; even if some low-quality data is inadvertently included in the analysis, the weights may mitigate the negative effects to some extent. Of course, as weights alone do not perfectly eliminate low-quality datasets (e.g., animal $\#20$), it is also important to investigate the details of the data and to be aware of low-quality animals by visual inspection. To assist such efforts, we have implemented several visualization functions (see Implementation).

## Discussion

In this analysis, we applied the WormTensor method to our whole-brain activity data from *C. elegans* and successfully obtained functional modules. In *C. elegans*, it is well known that most neurons do not have action potentials, and the neural activities instead change gradually. In addition, the temporal patterns of neural activities show positive and negative correlations with each other as well as lags of several lengths. Many biological dynamic systems, spanning intracellular signal transduction to animal behaviors, have similar features, and WormTensor should be suitable for extracting functional modules from temporal dynamics of such systems.

**Fig. 7:**
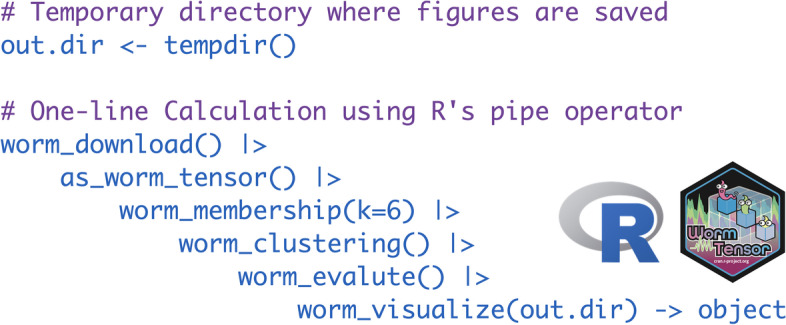
Workflow of the WormTensor package. All the functions can be performed using R’s native pipe operation

Because we did not set the upper and lower limits of the lag, it may be reasonably speculated many false positive pairs are clustered with extremely large (or small) ${\tau }$ values that are biologically meaningless. However, we concluded that such a bias is not severe in the clustering results. For details, see Additional file 13.

Because WormTensor uses mSBD, which treats correlation and anti-correlation equally by evaluating an absolute value function, one could suppose that PC1_pos and PC1_neg could be assigned a common cluster. However, we concluded that these two groups did not and should not be in the same cluster. For details, see Additional file 14.

Prior to the use of silhouette coefficients, we used various external and internal measures [18, 19] (all of those measures can be reproduced as functions inside WormTensor). However, for the present data, we chose not to use them, except for the silhouette coefficient per cell (Fig. 2). For the details, see Additional file 15.

By analyzing the neuronal activity data as a tensor instead of a matrix, the memory usage is increased from $\mathcal {O}\left( N \times N\right)$ to $\mathcal {O}\left( N \times N\times M\right)$, where *N* is the number of cells and *M* is the number of animals. Therefore, compared to matrix-based methods such as CSPA, WormTensor might be considered somewhat computationally disadvantageous. However, this is not much of a problem in realistic situations, that is, the number of somatic cells in *C. elegans* is approximately 1000 [20, 21], so the upper limit of *N* is fixed. Additionally, the data size does not become particularly large unless *M* is quite large (e.g., over 1000 animals).

We believe that the advantages of using tensor decomposition outweigh the above disadvantages; this approach not only improves silhouette coefficient compared to consensus clustering, as discussed in this work, but also allows for automatic estimation of weights. In data analysis that deals with multiple data sets (e.g., multiple individuals/multi-view/multi-modal/multi-omics data [22–26] and heterogeneous data fusion [27]), data with large values, a large number of patterns, and large size may dominate in optimization, and multiple data may not be treated equally. Alternatively, it may be the case that data of low quality are excluded from optimization because merging them with equal weight would reduce the performance of the overall optimization. In either case, it is necessary to appropriately weigh the individual datasets during optimization. Hence, weighting is not a trivial issue.

Some ad hoc weighting approaches, such as using L2-norm [23], the number of observed elements [28, 29], variance [25], the first eigenvalue [24], and correlations with an external standard [26, 30], can be used as the weight of each matrix/tensor, but it is unclear which approaches are appropriate. In contrast, in the tensor decomposition algorithm we used, the weights are not pre-set but are instead automatically estimated from the dataset. Therefore, there is no need to discuss the arbitrariness of the approaches described above. Moreover, based on the estimated weights, it can be appropriately used to investigate which animals were considered important or not. This ability is considered an excellent property for the purpose of QC.

From a different point of view, outlier animals with clusters that are not common among multiple animals might be biologically meaningful. Although the original purpose of WormTensor is to find clusters that are common among multiple animals and the weights can be interpreted as the degree of commonality, since a small weight means that the data of the animal is not similar to those of other animals, the weights could also assist the task of finding outlier animals in the data. After finding the outliers, some analytical methods can be individually applied to such animals and we can investigate the details.

### Comparison with other studies

Several methods have been applied to analyze whole-brain neural activity in *C. elegans*. A switching linear dynamical system (SLDS) provides a method to estimate discrete hidden states in time series data. The SLDS method has been applied to whole-brain neural activity to estimate the internal states of neural networks common across multiple animals and their transitions [31]. Other reports have analyzed whole-brain neural activity using PCA [8], ridge regression [32], and maximum entropy models [33], but these have only been applied to single animals.

Tensor decomposition has been applied to analyze large-scale neural activity data. For example, tensor component analysis decomposes the neural population recordings of mice into neuronal, temporal, and trial factors and is able to extract gradual changes in neural activity as learning progresses [34]. As another example, the shifted CP method decomposes electroencephalography (EEG) data from human subjects as a spatiotemporal superposition of components with a fixed time course and correctly unmixes the spatiotemporal signals in the EEG data [35]. Thus, tensor decomposition is useful for analyzing large-scale neural activity data.

## Conclusion

Our analysis showed that the differences among distance measures and clustering algorithms can influence the results of time-series clustering the neuronal activity of *C. elegans*. The combination of mSBD and MC-MI-HOOI maximized the silhouette coefficients compared to the other tested combinations of methods and also matched well with prior knowledge about *C. elegans* neural modules.

To create distance matrices, we used Euclidean distance and mSBD, with the latter contributing to the detection of specific clusters of cell populations such as PC1_neg- and PC2-related cells. This suggests that the features considered only in mSBD, such as correlations in both positive and negative directions and lags, are important for detecting functional modules from *C. elegans* neural activity data.

The data in this study included some missing values in all animals, and when we assessed the only intersection of those cells with no missing values, not a single cell remained. In addition, it is difficult to determine how to make a comprehensive judgment when analyzing the data for each animal because of the degree of missing data and the large differences among the animals. Therefore, dealing with missing values was essential in this analysis. In our study, by simply setting the missing cells to 0 at the stage of creating the membership matrices, we were able to proceed with the subsequent analysis and extract biologically meaningful patterns.

Compared with CSPA, which takes the average of multiple clustering results into a consensus matrix, we found that the use of MC-MI-HOOI has some advantages; it does not use the average of clustering results, and it automatically assigns a weight to each animal instead while allowing for individual differences. In this work, the latter approach was empirically advantageous to detect functional modules from *C. elegans* neural activity data.

Furthermore, the estimated weights are expected to be useful in other analyses; MC-MI-HOOI automatically avoids clustering results derived from noisy data by assigning small weights to the corresponding animals, and the weights themselves can reasonably be used for QC of data from animals.

All of the analyses including time-series clustering and visualizations performed in this work have been implemented as R functions available within the WormTensor R/CRAN package and can be freely reproduced with user data.

## Materials and methods

### Dataset

The whole-brain activity dataset of *C. elegans* strains JN3038 obtained by Toyoshima et al. [6] was used in this study. Briefly, each adult animal was held in a custom microfluidic chip and repeatedly stimulated by switching the sodium chloride concentration between 50mM and 25mM every 30 seconds. The volumetric movie of the head region of the animal was recorded at approximately 5 volumes per second using customized spinning-disc confocal microscopy. Genetically encoded calcium indicator Yellow-Cameleon 2.60 [36] was expressed in all neurons of the animals. For each neuron, the time-series of fluorescent intensities of yellow fluorescent protein (YFP) and cyan fluorescent protein (CFP) of Yellow-Cameleon were obtained from the volumetric movie. The intensity ratio of YFP over CFP indicates the calcium level (fluorescence resonance energy transfer; FRET [36]) and was used as an index of neural activity. A median filter [37] with an 11-time point window was applied to the ratio to remove noise. The outlier neurons were removed from the dataset if the filtered ratio of the neuron contained missing values, negative values, or values larger than 10. The filtered ratio was smoothed using a third-order Savitzky–Golay filter [38, 39] with a 101-time-point window. Finally, the time-series of smoothed ratios were scaled by dividing them by the mean value and subtracting 1. Annotation of neuronal identity was performed based on the spatial expression patterns of cell-specific promoters (i.e., landmark fluorescence) in the JN3038 strain. The dataset contains several non-neuronal cells, including GLR glial cells, pharyngeal gland cells, and hypodermal cells. Because a hypodermal cell has multiple nuclei (i.e., syncytium) and our experimental setup can detect each nucleus, we labeled them with the original name starting with HYPL.

The time-series neural activity values obtained from each animal were stored as a cell $\times$ time matrix. The measurements were collected from 28 animals, 4 of which were removed by QC, and the remaining 24 animals were used for our analysis (Fig. 1a and Table 2). Here, 192 cells in the 24 animals for which the neural activity values were measured at least once were included in the analysis. All neural activity values were measured for 6, 000 frames and the sampling rate (frame/sec) ranges from 3.69 to 5.73 (Table 2). The above data matrices were converted to distance matrices (Fig. 1a). In this work, we used two distance measures: Euclidean distance and modified shape-based distance (mSBD). We explain mSBD below.

#### Modified shape-based distance

To consider the lags of cell–cell interactions between neurons, we used a shift-invariant distance measure called shape-based distance (SBD [40]) with some modification. For two arbitrary time-series data *x* and *y* SBD is given as

1 $$\begin{aligned} \textrm{SBD}\left( x,y\right) = 1 - \max _{\mathrm {\tau }}{\left( \frac{R_{\tau -m}\left( x, y\right) }{\sqrt{\textrm{R}_{0}\left( x,x\right) \textrm{R}_{0}\left( y,y\right) }}\right) }, \end{aligned}$$

where $\tau \in \{1,2,...,2m-1\}$ is lag when sliding *x* to the right and *m* is the length of *x* and *y*. $R_{\tau -m}\left( x, y\right)$ is the cross-correlation measure between *x* and *y* with lag $\tau$. $R_{\tau -m}\left( x, y\right)$, $\textrm{R}_{0}\left( x,x\right)$, and $\textrm{R}_{0}\left( x,x\right)$ are calculated as the inner product between *x* and *y* with lag $\tau$ as follows:

2 $$\begin{aligned} R_{\tau }\left( x,y\right) = {\left\{ \begin{array}{ll} {\sum }_{l=1}^{m-k}{x_{l+\tau } \cdot y_{l}} &{} \left( \tau \ge 0\right) \\ R_{-\tau }\left( y,x\right) &{} \left( \tau < 0\right) \end{array}\right. }. \end{aligned}$$

To achieve shift-invariance, cross-correlation keeps *y* static and slides *x* over *y* to compute their inner product for each shift $\tau$ of *x*. When sliding *x*, only the elements of *x* and *y* that share time are used to calculate cross-correlation. To capture the negative correlation between neurons, we defined mSBD as

3 $$\begin{aligned} {\textrm{mSBD}}\left( x,y\right) = 1 - \max _{\tau }{\left( \textrm{abs}{\left( \frac{R_{s-m}\left( x, y\right) }{\sqrt{\textrm{R}_{0}\left( x,x\right) \textrm{R}_{0}\left( y,y\right) }}\right) }\right) }, \end{aligned}$$

where the absolute value function $abs\left( \right)$ is included to search for the highest correlation. This makes it possible to handle the interactions between cell pairs with high positive correlation such as AVAR and RIMR (Fig. 1b), and cell pairs with high negative correlation such as ASEL and ASER (Fig. 1c), in a unified manner. This modification is based on mutual inhibition, in which the A-type and B-type command interneurons inhibit each other, resulting in a negative correlation between these neurons [41].

### Membership matrices

For each distance matrix by mSBD (Eq. (3), hierarchical clustering (Ward’s method [42]) with *K* clusters was applied, and based on the results, we obtained a binary matrix (membership matrix) (Fig. 1a). The membership matrix of the *m*-th animal is defined as follows:

4 $$\begin{aligned} \left[ S_{m}\right] _{i,j} = {\left\{ \begin{array}{ll} 1 &{} \left( \text {if cell }i\text { and cell }j\text { belong to the same cluster}\right) \ \\ 0 &{} \left( \text {otherwise}\right) \end{array}\right. }. \end{aligned}$$

If a cell was not identified in an animal, it was assumed that the cell did not belong to the same cluster as any of the other cells.

### Clustering

#### Cluster-based similarity partitioning

Consensus clustering (or cluster ensembles) was performed on the membership matrices described above. Note that consensus clustering is the generic term for clustering algorithms that aggregate multiple results of clustering. In this work, we used CSPA, which is perhaps the simplest and most widely used consensus clustering algorithm [11, 12].

In CSPA, the consensus matrix $S \in \mathbb {R}^{N \times N}$ (Fig. 1a) is calculated by averaging all the membership matrices as follows:

5 $$\begin{aligned} \left[ S\right] _{i,j} = \frac{1}{M}\sum _{m=1}^{M}{\left[ D_{m}\right] _{i,j}}. \end{aligned}$$

Here, *S* is then converted to a dissimilarity matrix by $1-S$ and used for further analysis such as clustering or dimensionality reduction by t-SNE and UMAP. For clustering, we used Ward’s hierarchical clustering with *K* clusters, which is the same number of clusters we set when we created the membership matrices above.

#### WormTensor

WormTensor performs two main processes. First, it uses mSBD described above to account for the lags and the mutual inhibition of cell–cell interactions. Second, it uses tensor decomposition for clustering to weigh each animal without averaging among them. Unlike CSPA, which reduces multiple membership matrices into a matrix *S*, WormTensor stacks the *M* membership matrices in the depth direction (the 3-rd dimension) and creates a third-order tensor $\mathcal {X} \in \mathbb {R}^{N \times N \times M}$ (Fig. 1a).

In WormTensor, the tensor decomposition algorithm MC-MI-HOOI is applied to this tensor. MC-MI-HOOI performs decomposition as follows:

6 $$\begin{aligned} \left[ \mathcal {X}\right] _{::m} \approx w_{m} U G U^{T}. \end{aligned}$$

Alternatively, in the matrix/tensor form, this is formalized as

7 $$\begin{aligned} \mathcal {X} \approx G \times _{1} U \times _{2} U \times _{3} W, \end{aligned}$$

where $U \in \mathbb {R}^{N \times K}$ is the factor matrix, $G \in \mathbb {R}^{K \times K}$ is the core tensor (a diagonal matrix), each element $w_{m}$ of vector $W \in \mathbb {R}^{M}$ is the weight of *m*-th slice of $\mathcal {X}$, and $\times _{l}$ is the mode-*l* product [43].

To decompose *U*, *G*, and *W*, MC-MI-HOOI solves the following optimization problem:

8 $$\begin{aligned} \begin{aligned} \max _{U,w}{\Vert U^{T} \left( \sum _{k=1}^{K}{w_{k}} \left[ \mathcal {X}\right] _{::k} \right) U \Vert _{F}^{2}} \\ \text {s.t.} \, U^{T}U=I_{K},\, \Vert W \Vert _{F}^{2} = 1 \end{aligned}. \end{aligned}$$

Alternatively, in the matrix/tensor form, this is formalized as

9 $$\begin{aligned} \begin{aligned} \max _{U,w}{\Vert \mathcal {X} \times _{1} U^{T} \times _{2} U^{T} \times _{3} W^{T}\Vert _{F}^{2}} \\ \text {s.t.} \, U^{T}U=I_{K},\, \Vert W \Vert _{F}^{2} = 1 \end{aligned}, \end{aligned}$$

where $I_{K} \in \mathbb {R}^{K \times K}$ is the identity matrix of size *K*.

Because MC-MI-HOOI is a special case of *higher orthogonal iteration of tensors* (HOOI [43]), which is an algorithm that is widely used to solve Tucker decomposition ($\mathcal {X} \approx G \times _{1} A_{1} \times _{2} A_{2} \times _{3} A_{3}$), we obtained *U* and *W* of MC-MI-HOOI via HOOI by setting the dimension of the depth factor matrix $A_{3}$ to 1, assuming that the first and second factor matrices ($A_{1}$ and $A_{2}$) are common. After HOOI converges, *G* is calculated as $G = \mathcal {X} \times _{1} U^{T} \times _{2} U^{T} \times _{3} W^{T}$.

After the optimization, *U* can be used for further analysis such as clustering or dimensional reduction by t-SNE and UMAP. To perform these analyzes, we first created the distance matrix of *U*. For clustering, we used Ward’s hierarchical clustering with *K* clusters, which is the same number of clusters we set when we created the membership matrices above.

#### Silhouette coefficient

To estimate the number of clusters to be used by CSPA and WormTensor, we used the silhouette coefficient [13]. To obtain the silhouette coefficient for a cell *i* belonging to a cluster $C_{a}$, the average distance between cell *i* and the other cells in $C_{a}$ $a\left( i \right)$ is calculated as

10 $$\begin{aligned} a\left( i \right) = \frac{1}{|C_{a}|-1}\sum _{j \in C_{a}, i \ne j}{d\left( i,j\right) }, \end{aligned}$$

where $|C_{a}|$ is the number of cluster members in $C_{a}$ and $d\left( i,j\right)$ is the distance between cell *i* and cell *j*. Then, the minimum average distance between cell *i* and the other cells in $C_{b} \left( C_{b} \ne C_{a}\right)$ $b\left( i \right)$ is calculated as follows:

11 $$\begin{aligned} b\left( i \right) = \min _{C_{b} \ne C_{a}}\frac{1}{|C_{b}|}\sum _{j \in C_{b}}{d\left( i,j\right) }. \end{aligned}$$

Finally, the silhouette coefficient for cell *i* $\textrm{s}\left( i \right)$ is obtained as follows:

12 $$\begin{aligned} \textrm{s}\left( i \right) = \frac{b\left( i \right) - a\left( i \right) }{\max {\left\{ a\left( i \right) , b\left( i \right) \right\} }}. \end{aligned}$$

The value spans $-1$ to 1, and the closer the value is to 1, the more aggregated the cells are with those belonging to the same cluster as cell *i*. Conversely, a value close to $-1$ means that there are cells belonging to other clusters in the neighborhood of cell *i*, which means that the clustering did not perform well.

#### Consistency

To interpret the results of clustering by WormTensor and CSPA, we defined the consistency between the clusters of the merged animals (WormTensor or CSPA) and the clusters in each animal. We defined the consistency of cell *i* ($\textrm{c}_{i}$) as

13 $$\begin{aligned} \textrm{c} \left( i \right) = {\left\{ \begin{array}{ll} {\sum }_{m=1}^{M}{\frac{|C_{\textrm{merged}} \cap C_{m}| - 1}{|C_{\textrm{merged}}| - 1}} &{} (C_{\textrm{merged}} \ne C_{\textrm{m}})\\ 0 &{} (C_{\textrm{merged}} = C_{\textrm{m}}), \end{array}\right. } \end{aligned}$$

where $i \in C_{\textrm{merged}} \cap C_{m}$, *M* is the number of animals, $C_{\textrm{merged}}$ is the group of members of the cluster of the merged animals, and $C_{\textrm{m}}$ is the group of members of the cluster of the *m*-th animal. The value of $\textrm{c}_{i}$ ranges from 0 to *M*.

#### Adjusted rand index

To evaluate the similarity of the clustering results of WormTensor and the clustering results of individual animals, we utilized ARI [17], which is an external validity index that is widely used to evaluate clustering results with known class labels.

Suppose that *N* cells are divided into *K* clusters by WormTensor such that $C = \{C_{1}, C_{2},..., C_{K}\}$. Likewise, suppose that *N* cells are divided into $K'$ clusters by Ward’s hierarchical clustering in an animal such that $C' = \{C'_{1}, C'_{2},..., C'_{K'}\}$. From these two partitions, the Rand index (RI) is defined as a similarity measure between two partitions as

14 $$\begin{aligned} {{\textrm{RI}}} = \frac{ \left( a + d \right) }{ \left( a + b + c + d \right) }, \end{aligned}$$

where *a* is the number of pairs within *N* cells that are in the same subset in *C* and in the same subset in $C'$, *b* is the number of pairs within *N* cells that are in different subsets in *C* and in different subsets in $C'$, *c* is the number of pairs within *N* cells that are in the same subset in *C* and in different subsets in $C'$, and *d* is the number of pairs within *N* cells that are in different subsets in *C* and in the same subset in $C'$.

ARI is an adjusted version of RI that corrects the bias towards RI increasing by chance.

15 $$\begin{aligned} {\textrm{ARI}} = \frac{ {\textrm{RI}} - \textrm{E} \left( {\textrm{RI}} \right) }{ \max {\left( {\textrm{RI}}\right) } - \textrm{E} \left( {\textrm{RI}} \right) , } \end{aligned}$$

where $\textrm{E} \left( {\textrm{RI}} \right)$ is the expected value of RI based on some probability distribution. In this work, we used the permutation model based on the generalized hypergeometric distribution [17], which is the most widely used model for ARI. RI and ARI range from 0 to 1, with higher values indicating a better match between the WormTensor clustering results and the clustering results in each animal.

### Implementation

The WormTensor package is implemented in R and is made available through CRAN (https://cran.r-project.org) under the MIT license. WormTensor consists of seven R functions described below.

- worm_download() downloads the distance matrices used in this work from figshare; 28 animals’ data, including those of 24 used in this study and 4 noisy ones, are available (Table 2).
- worm_distance() generates the distance matrices between cells for multiple animals from input time-series data matrices (cells $\times$ time) specified by users; mSBD, SBD, or Euclidean distance can be specified as the distance measure (default, mSBD).
- as_worm_tensor() instantiates a WormTensor object from the distance matrices, which are used in the following functions.
- worm_membership() creates a membership tensor from the results of clustering performed using the distance matrix of each animal.
- worm_clustering() performs clustering using the distance matrices above. MC-MI-HOOI and CSPA can be specified as the clustering algorithm (default, MC-MI-HOOI).
- worm_evaluate() evaluates the results of worm_clustering(). As internal validity indices [18, 19] without prior knowledge of the clusters, entropy, pseudo-F measure, and connectivity computations are implemented. As external validity indices [18, 19] using prior knowledge of the clusters, ARI, purity, and micro-averaged F-measure computations are implemented. The latter indices are optional, and only if the class label is specified, these indices are calculated.
- worm_visualize() visualizes the results of worm_clustering(). In addition, the number of identified cells and consistency are visualized as QC metrics. Only if some labels to interpret the clusters are specified (e.g., neuron type or neuron class), such labels are also visualized. Only if the algorithm of worm_clustering() is specified as MC-MI-HOOI, the relationship of the weights and the number of identified cells and ARI between the clustering result of each animal and the result of MC-MI-HOOI are visualized.

Inspired by the Tidyverse [44], WormTensor also uses R’s native pipe operator to allow multiple R functions to be chained together and executed as one-liner code (Fig. 7).

## Availability and requirements

### R/CRAN package

- WormTensor: https://cran.r-project.org/web/packages/WormTensor
- Operating system: Linux, Mac OS X, Windows
- Programming language: R (v$-$4.1.0 or higher)
- License: MIT
- Any restrictions to use by non-academics: For non-profit use only

### Docker container of WormTensor

- WormTensor: https://hub.docker.com/r/yamaken37/wormtensor

### Snakemake workflow

- WormTimeSeries (to fully reproduce the analyses in this study): https://github.com/rikenbit/WormTimeSeries
- Operating system: Linux, Mac OS X, Windows
- Programming language: Python (v$-$3.7.8 or higher), Snakemake (v$-$6.0.5 or higher), Singularity (v$-$3.8.0 or higher)
- License: MIT
- Any restrictions to use by non-academics: For non-profit use only

## Supplementary information

**Additional file 1.** Cellular labels for interpreting the clustering results.CSV 10.73 KB, https://figshare.com/ndownloader/files/38554109.

**Additional file 2.** Cellular labels for interpreting the clustering results).XLSX 233.53 KB, https://figshare.com/ndownloader/files/38554106.

**Additional file 3.** WormTensor, the results of clusters + consistency + No. of cells.PNG 1.22 MB, https://figshare.com/ndownloader/files/36186645.

**Additional file 4.** WormTensor, neuron type + consistency + No. of cells.PNG 1.18 MB, https://figshare.com/ndownloader/files/36186651.

**Additional file 5.** WormTensor, class label + consistency + No. of cells.PNG 1.11 MB, https://figshare.com/ndownloader/files/36186669.

**Additional file 6.** MC-MI-HOOI with Euclidean distance, *k* = 10, the results of clusters + consistency + No. of cells.PNG 2.33 MB, https://figshare.com/ndownloader/files/36186681.

**Additional file 7.** MC-MI-HOOI with Euclidean distance, *k* = 10, neuron type + consistency + No. of cells.PNG 2.08 MB, https://figshare.com/ndownloader/files/36186693.

**Additional file 8.** MC-MI-HOOI with Euclidean distance, *k* = 10, class label + consistency + No. of cells.PNG 1.93 MB, https://figshare.com/ndownloader/files/36186714.

**Additional file 9.** Results of clustering with the optimal number of clusters. **a** The clustering results of CSPA with Euclidean distance. **b** The clustering results of MC-MI-HOOI with Euclidean distance. **c** The clustering results of CSPA with mSBD. **d** The clustering results of MC-MI-HOOI with mSBD.PNG 847.16 KB, https://figshare.com/ndownloader/files/36189936.

**Additional file 10.** Results of WormTensor. **a** Neuron type. **b** The labels of the movement of *C. elegans*. **c** Consistency between the results of hierarchical clustering in each animal and WormTensor. **d** The number of identified cells.PNG 869.43 KB, https://figshare.com/ndownloader/files/36189957.

**Additional file 11.** Hypergeometric test p-values and FDR *q*-values of all the clustering methods with the optimal numbers of clusters.XLSX 19.08 KB, https://figshare.com/ndownloader/files/38554103.

**Additional file 12.** **a** The order of WormTensor weights of the animals when animal 20 was added. **b** The order of WormTensor weights of the animals when four noisy datasets were added. **c** The abnormal waveforms of animal 20.PNG 416.57 KB, https://figshare.com/ndownloader/files/36619650.

**Additional file 13.** Relationship between the shift value and the maximum absolute correlation coefficient in mSBD.PDF 648.84 KB, https://figshare.com/ndownloader/files/38554100.

**Additional file 14.** Differences in the properties of PC1_pos- and PC1_neg-related cells.PDF 2.38 MB, https://figshare.com/ndownloader/files/38554097.

**Additional file 15.** Results for clustering evaluation measures other than silhouette coefficient.PDF 1.68 MB, https://figshare.com/ndownloader/files/38943455.

## Data Availability

All distance matrices analyzed during this study are included in this published article. The datasets of raw time series of neural activity will be available from a DOI (10.6084/m9.figshare.21968078) reserved for publication by Figshare.

## Abbreviations

*C. elegans*: $$Caenorhabditis\ elegans$$
SBD: Shape-based distance
mSBD: Modified shape-based distance
HOOI: Higher orthogonal iteration of tensors
MC-MI-HOOI: Multi-view clustering based on matrix integration using the HOOI algorithm
CSPA: Cluster-based similarity partitioning algorithm
NaCl: Sodium chloride
t-SNE: T-distributed stochastic neighbor embedding
UMAP: Uniform manifold approximation and projection
QC: Quality control
TCA: Tensor components analysis
EEG: Electro-encephalography
YFP: Yellow fluorescent protein
CFP: Cyan fluorescent protein
FRET: Fluorescence resonance energy transfer
RI: Rand index
ARI: Adjusted rand index
