# Supplementary figures and images for: WormTensor: a clustering method for time-series whole-brain activity data from C. elegans
Source: BMC Bioinformatics. 2023 Jun 16;24:254. doi: 10.1186/s12859-023-05230-2 (PMC10273573; doi:10.1186/s12859-023-05230-2)

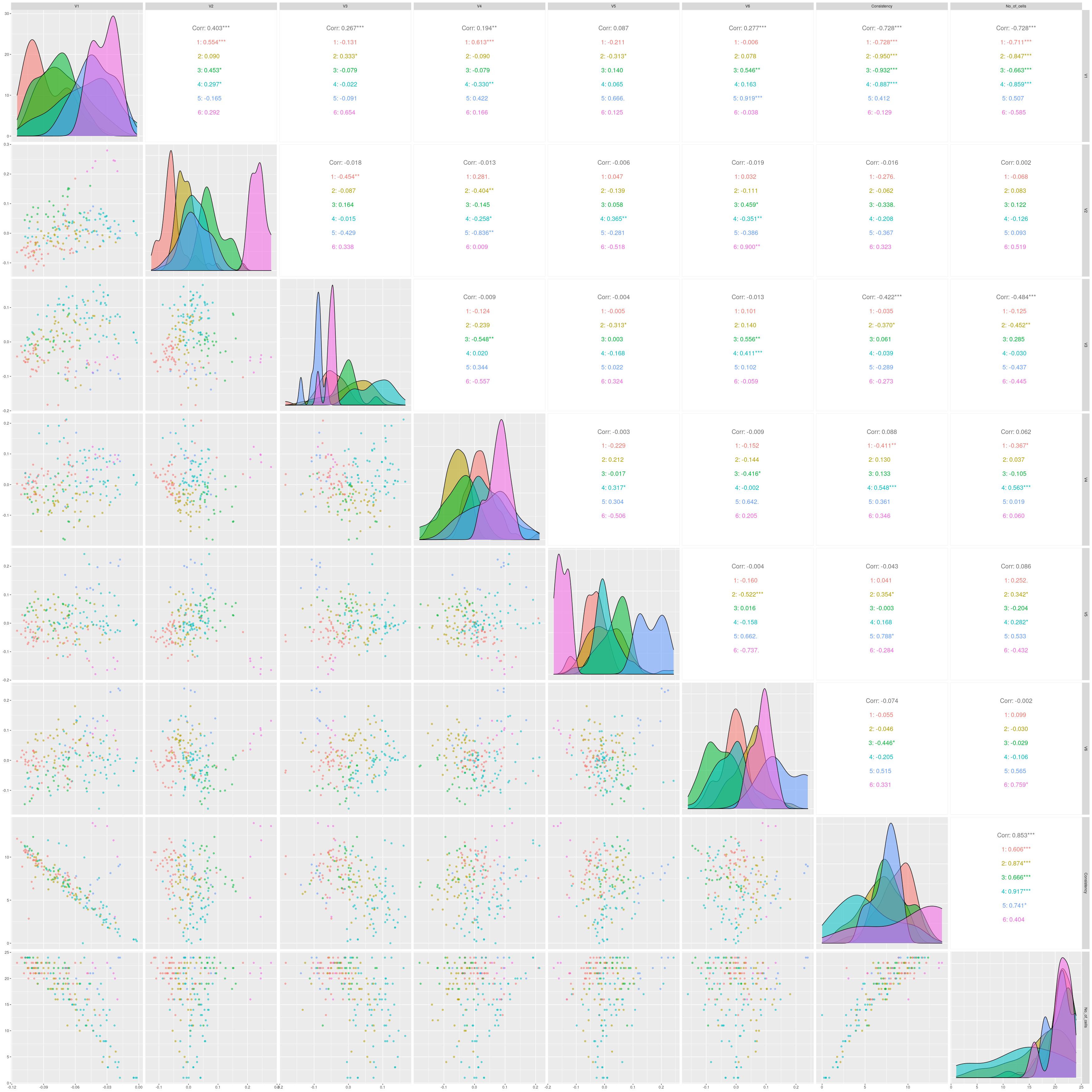

Supplement: Supplementary file 3 — Additional file 3. WormTensor, the results of clusters + consistency + No. of cells.PNG 1.22 MB, https://figshare.com/ndownloader/files/36186645. [file 12859_2023_5230_MOESM3_ESM.png]

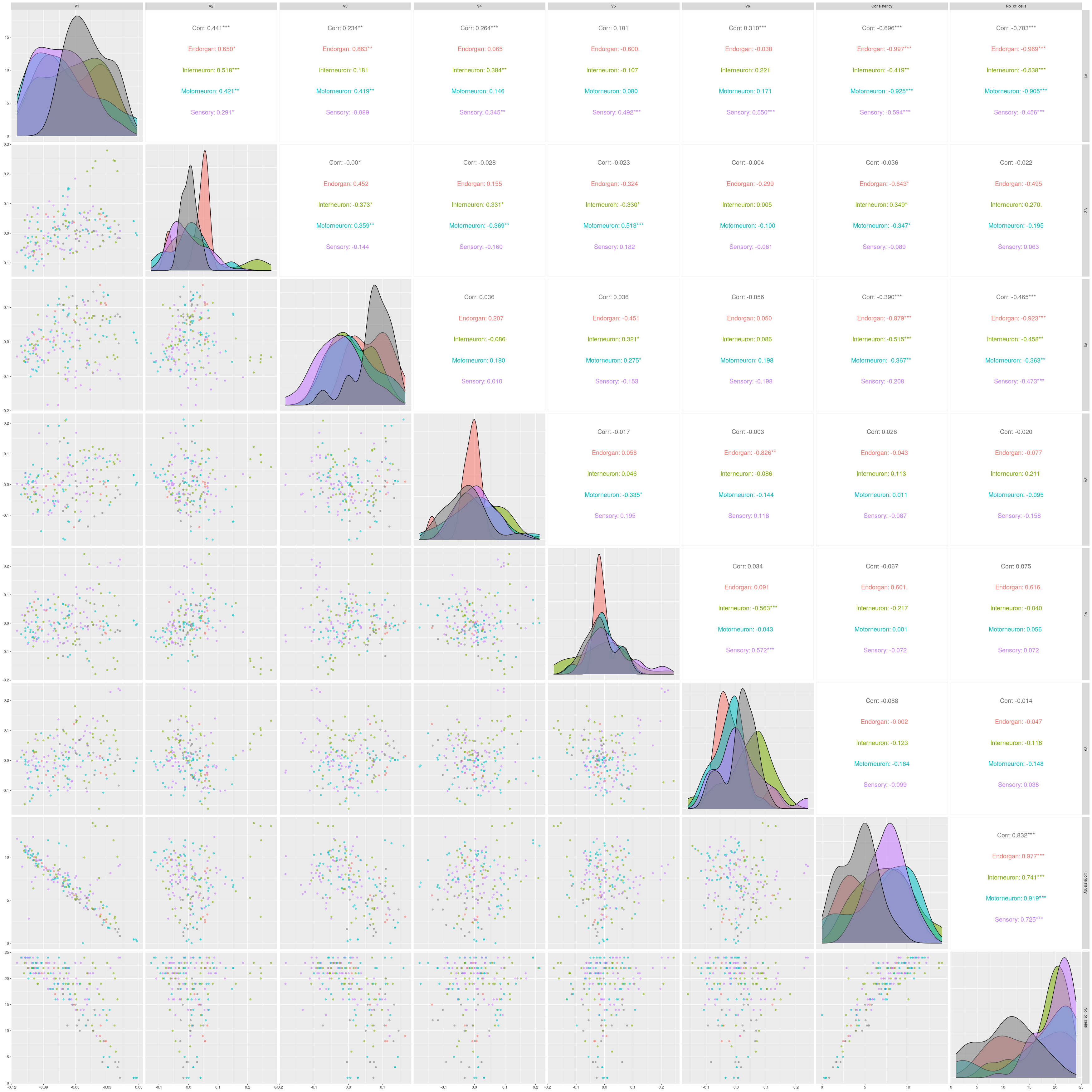

Supplement: Supplementary file 4 — Additional file 4. WormTensor, neuron type + consistency + No. of cells.PNG 1.18 MB, https://figshare.com/ndownloader/files/36186651. [file 12859_2023_5230_MOESM4_ESM.png]

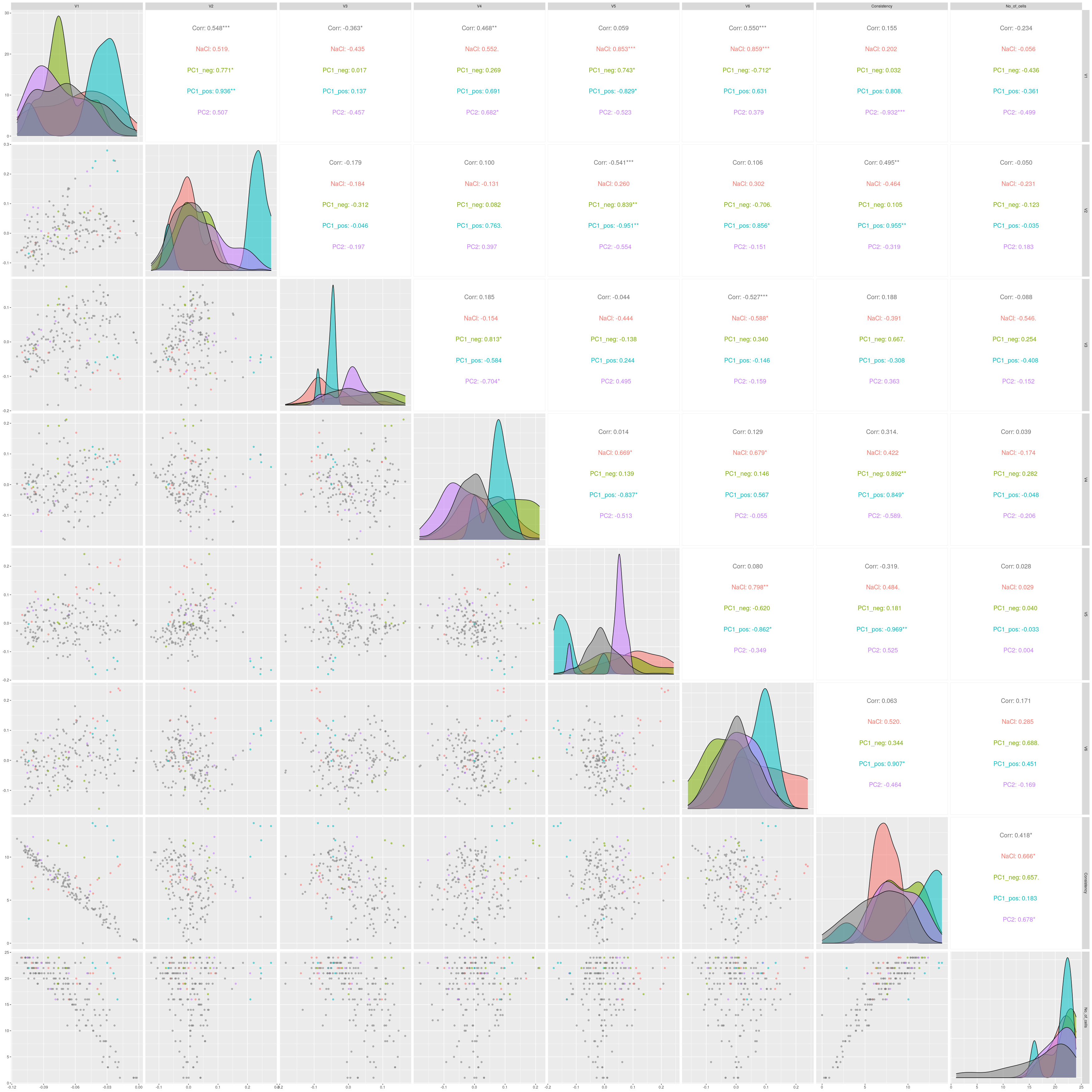

Supplement: Supplementary file 5 — Additional file 5. WormTensor, class label + consistency + No. of cells.PNG 1.11 MB, https://figshare.com/ndownloader/files/36186669. [file 12859_2023_5230_MOESM5_ESM.png]

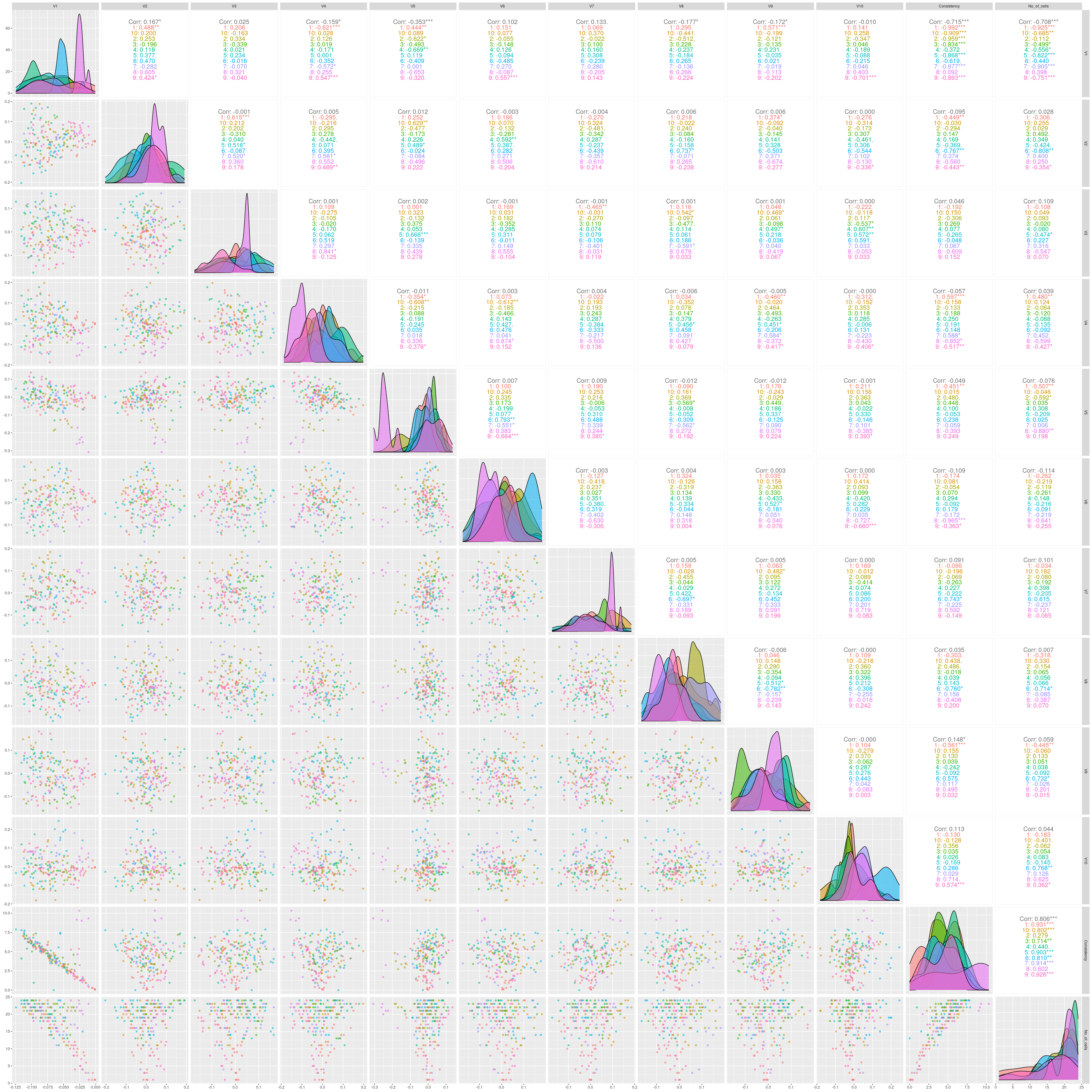

Supplement: Supplementary file 6 — Additional file 6. MC-MI-HOOI with Euclidean distance, k = 10, the results of clusters + consistency + No. of cells.PNG 2.33 MB, https://figshare.com/ndownloader/files/36186681. [file 12859_2023_5230_MOESM6_ESM.png]

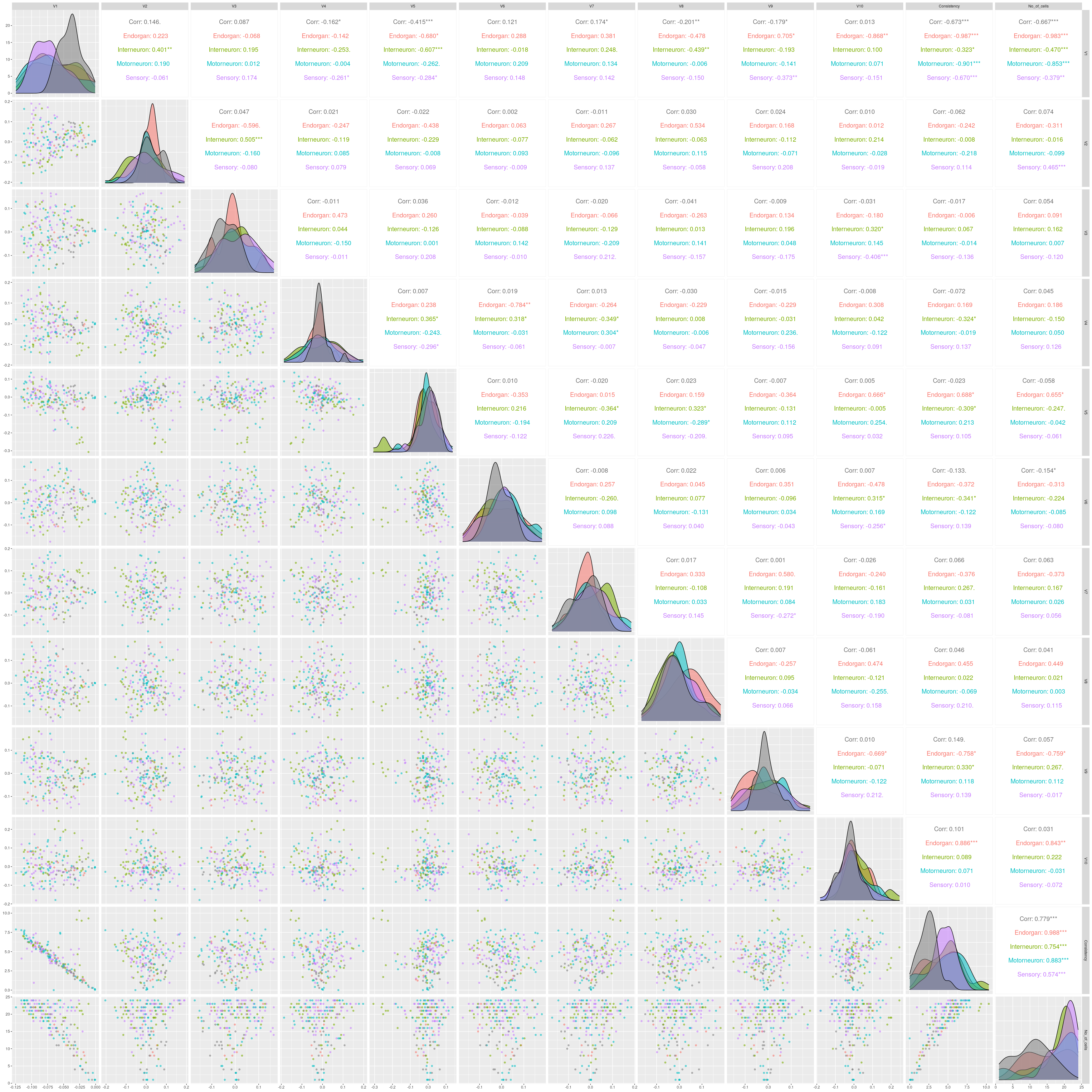

Supplement: Supplementary file 7 — Additional file 7. MC-MI-HOOI with Euclidean distance, k = 10, neuron type + consistency + No. of cells.PNG 2.08 MB, https://figshare.com/ndownloader/files/36186693. [file 12859_2023_5230_MOESM7_ESM.png]

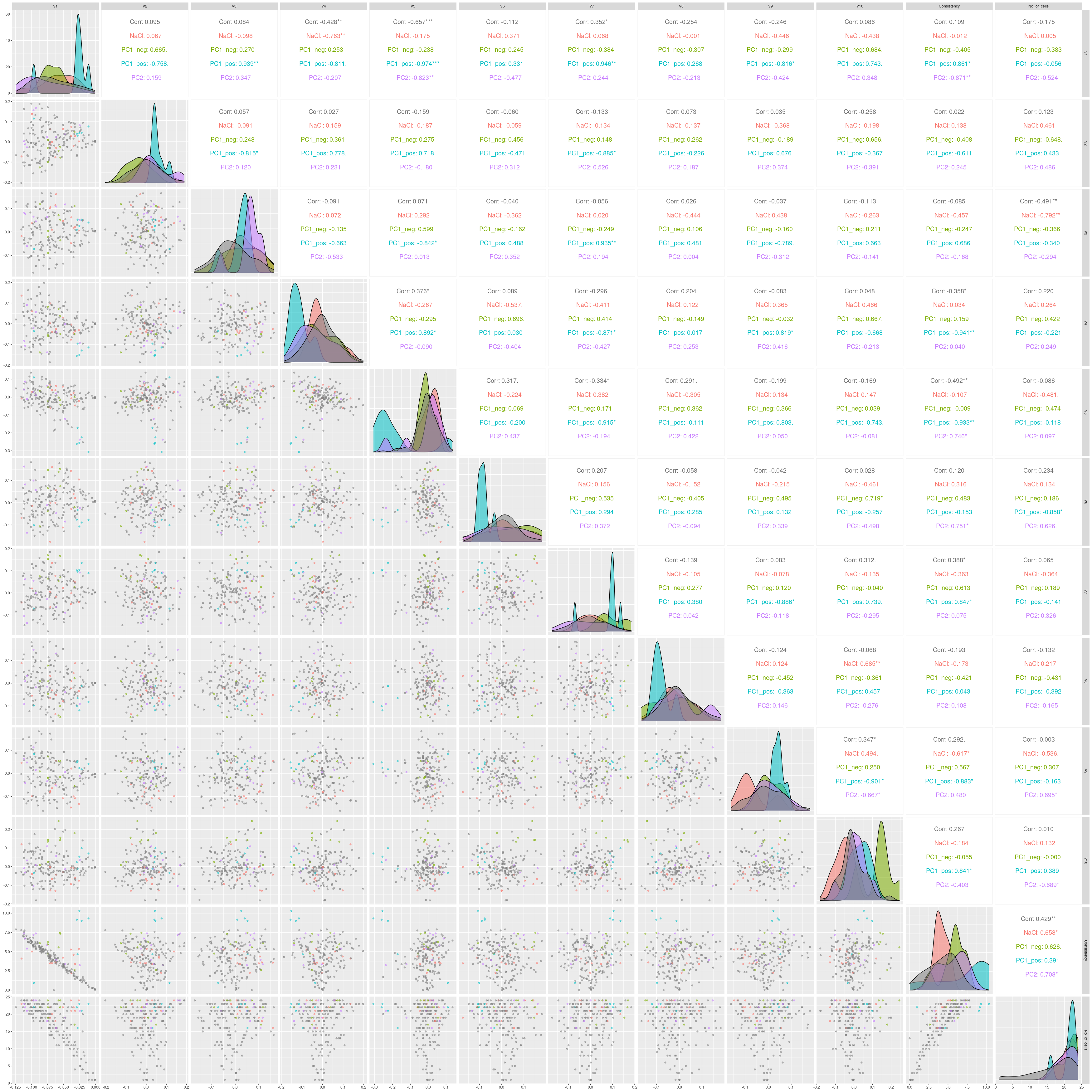

Supplement: Supplementary file 8 — Additional file 8. MC-MI-HOOI with Euclidean distance, k = 10, class label + consistency + No. of cells.PNG 1.93 MB, https://figshare.com/ndownloader/files/36186714. [file 12859_2023_5230_MOESM8_ESM.png]
