# Supplementary material for: WormTensor: a clustering method for time-series whole-brain activity data from C. elegans
Source: BMC Bioinformatics. 2023 Jun 16;24:254. doi: 10.1186/s12859-023-05230-2 (PMC10273573; doi:10.1186/s12859-023-05230-2)
Supplement: Supplementary file 13 — Additional file 13. Relationship between the shift value and the maximum absolute correlation coefficient in mSBD.PDF 648.84 KB, https://figshare.com/ndownloader/files/38554100. [file 12859_2023_5230_MOESM13_ESM.pdf]

Relationship between the shift value and the maximum absolute correlation coefficient in mSBD

In this work, we used modified shape-based distance (mSBD) to calculated the distance between all the cell pairs; mSBD applies a time shift between two time series vectors until the shift corresponds to the largest absolute correlation coefficient. Because we 'did not set the upper and lower limits of the shift, some researchers might reasonably wonder if many false positive pairs are clustered with extremely large (or small) shift value that are biologically meaningless. Here we confirmed that such a bias is not severe in the clustering results.

First, we summarized the shift values and the corresponding largest absolute correlation coefficient values (Figure S13-1). We found that the shift values automatically assigned by mSBD are enriched around the origin point. This was a common trend among the 24 animals used in this study (Figure S13-2).

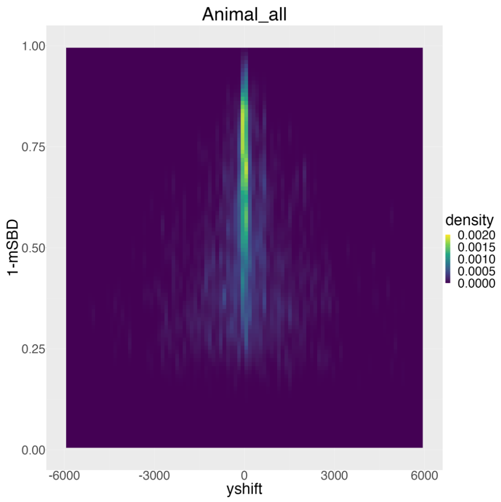

Figure S13-1 | 2D density distribution of the shift values and the largest absolute correlation coefficient values. The *x*- and *y*-axes indicate the shift values of mSBD and the largest absolute correlation coefficient values in the shift values, respectively.

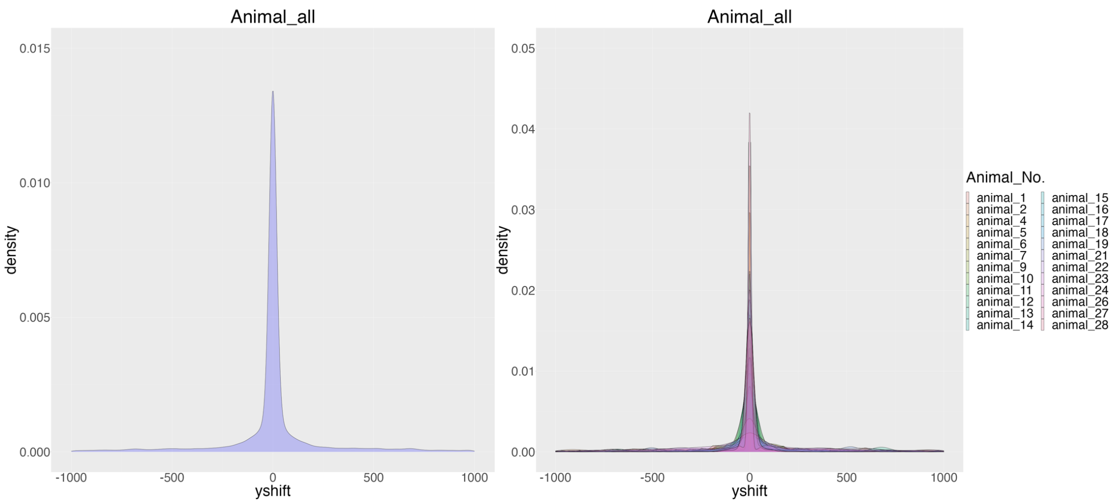

Figure S13-2 | Density distributions of the shift values (left) of all the animals together and (right) each animal individually.

It was also found that the largest absolute correlation coefficient values decreased as the shift values increased (Figure S13-3).

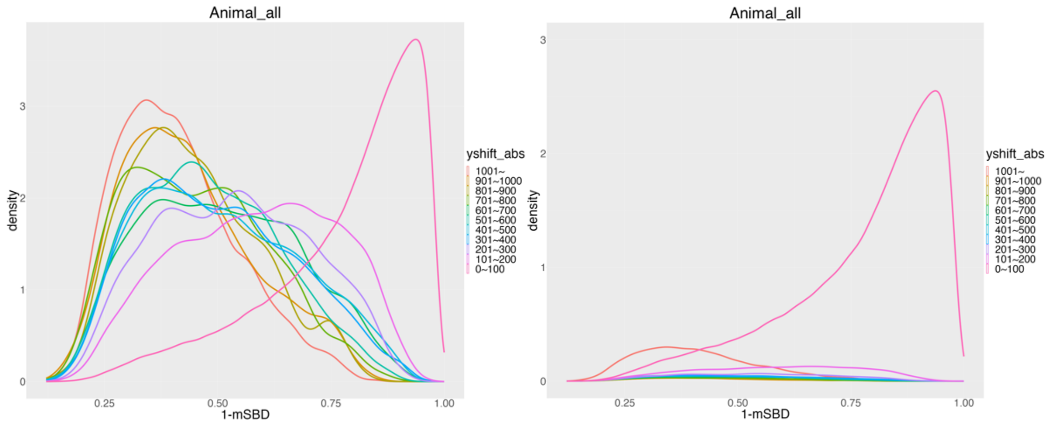

Figure S13-3 | Density distributions of the largest absolute correlation coefficient values of the shift values for data from all animals. The distributions are shown for the indicates ranges of absolute shift values. (Left) The areas of all the density distributions are the same. (Right) Each area of the density distribution is reflected by the frequency of the corresponding data for each absolute shift value range.

This analysis suggests that the cell pairs with large shift values are not likely to be clustered together because the low values of the largest absolute correlation coefficient (or high values of mSBD) mean these cells have dissimilar patterns. In contrast, the cell pairs with small shift values would be easily clustered together. As most of the shift

values with the largest absolute correlation coefficient values are enriched around the origin point, it is likely that the potential bias we considered was not severe and had little effect on the detection of false positive pairs with very large shift values.

Next, we investigated the possible difference in shift values between cell pairs clustered together and cell pairs not clustered together by hierarchical clustering. As we expected above, the shift values of the cell pairs clustered together actually had low variances (Table S13-1), and all the F-tests performed on analyses based on each animal were highly significant, compared with the shift values of the cell pairs not clustered together.

These results suggest that the shift values automatically selected by mSBD are enriched around the origin point and that even if the largest absolute correlation coefficient with an extremely large (or small) shift value is selected by mSBD, the mSBD value itself tends to be small, which means there is little effect on the detection of false positive clusters in hierarchical clustering.

Table S13-1 | Summary of the shift values of mSBD for data from each animal. Here, 2SD refers to the double standard deviation of the shift values, while 2SD (0) and 2SD (1) specifically refer to the cell pairs that were and were not clustered together, respectively. To test the statistical significance of variance between two groups (0 and 1), we performed unpaired F-tests (-$\log_{10}$(p-value) shown for each animal). To correct for the multiple testing problem, we also performed an FDR method (Benjamini-Hochberg) (-$\log_{10}$(q-value) shown for each animal).

Animal No.	2SD (0)	2SD (1)	2SD (all)	F-test (-$\log_{10}$(p-value))	F-test (-log10(q-value))
1	1097	344	901	15.7	15.2
2	1439	955	1347	15.7	15.2
4	1017	378	888	15.7	15.2
5	675	109	479	15.7	15.2
6	962	221	821	15.7	15.2
7	855	412	753	15.7	15.2
9	598	135	518	15.7	15.4
10	475	47	334	15.7	15.2
11	962	95	660	15.7	15.2
12	928	340	796	15.7	15.2
13	485	199	395	15.7	15.2
14	230	66	153	15.7	15.5
15	703	267	620	15.7	15.6
16	344	52	269	15.7	15.2
17	412	48	327	15.7	15.5
18	787	309	662	15.7	15.5
19	841	239	717	15.7	15.5
21	1196	635	1096	15.7	15.2
22	818	595	785	15.7	15.2
23	817	557	761	15.7	15.2
24	411	47	309	15.7	15.5
26	272	71	187	15.7	15.2
27	190	173	184	15.4	14.9
28	154	8	121	15.7	15.2
