# Supplementary material for: WormTensor: a clustering method for time-series whole-brain activity data from C. elegans
Source: BMC Bioinformatics. 2023 Jun 16;24:254. doi: 10.1186/s12859-023-05230-2 (PMC10273573; doi:10.1186/s12859-023-05230-2)
Supplement: Supplementary file 14 — Additional file 14. Differences in the properties of PC1_pos- and PC1_neg-related cells.PDF 2.38 MB, https://figshare.com/ndownloader/files/38554097. [file 12859_2023_5230_MOESM14_ESM.pdf]

Differences in the properties of PC1_pos- and PC1_neg-related cells

In this work, we used cellular labels indicating the known functional modules as defined by Saul Kato *et al.* [1] to evaluate the accuracy of clustering. In their study, principal component 1 (PC1)-related cells were also separated into $PC1_{pos}$- and $PC1_{neg}$-related cells based on the sign of coefficients. In our analysis, $PC1_{pos}$-related cells were clustered in a common cluster, but $PC1_{neg}$-related cells were scattered across many clusters. Because *WormTensor* uses mSBD, which treats correlation and anti-correlation equally according to the absolute value function, one might think that $PC1_{pos}$- and $PC1_{neg}$-related cells could be assigned to a common cluster. We found the following reasons they were assigned to different clusters.

First, we found that the maximum absolute correlation coefficient values between $PC1_{pos}$ and $PC1_{neg}$ were lower than that of $PC1_{pos}$ and $PC1_{pos}$ or $PC1_{neg}$ and $PC1_{neg}$ (Figure S14-1 and S14-2). We also found that the shift values between $PC1_{pos}$ and $PC1_{neg}$ were higher than that of $PC1_{pos}$ and $PC1_{pos}$ or $PC1_{neg}$ and $PC1_{neg}$ (Figures S14-3 and S14-4). This tendency is consistent with the fact that the absolute correlation coefficient and the time shift value are anti-correlated in mSBD (Additional File 13). Consequently, $PC1_{pos}$-related cells and $PC1_{neg}$-related cells are unlikely to be in the same cluster.

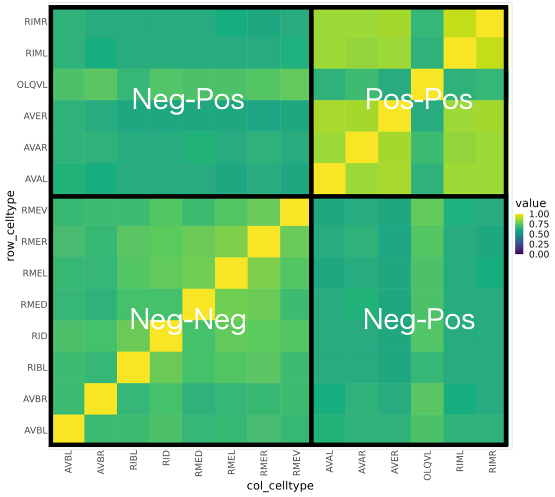

Figure S14-1 | The largest absolute correlation coefficient between two cells (across all animals). Neg refers to $PC1_{neg}$-related cells, and Pos refers to $PC1_{pos}$-related

cells.

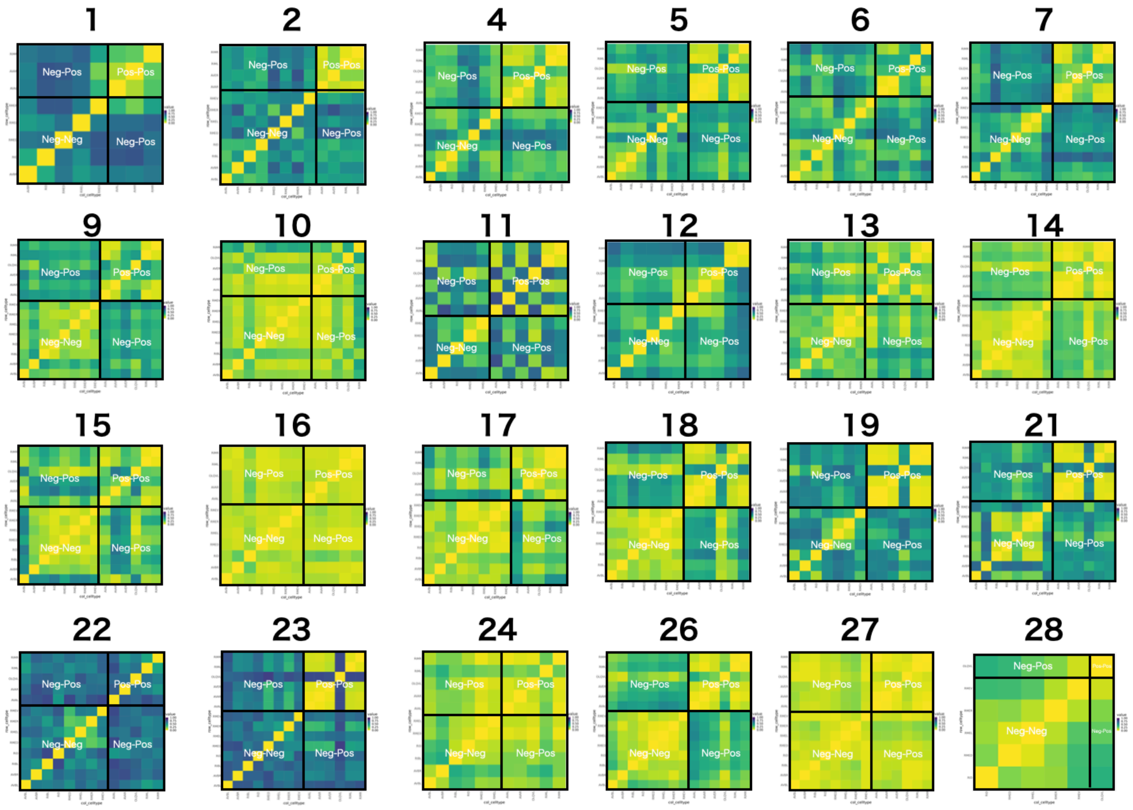

Figure S14-2 | The largest absolute correlation coefficient between two cells (within each animal). Neg refers to PC1$_{neg}$-related cells, and Pos refers to PC1$_{pos}$-related cells.

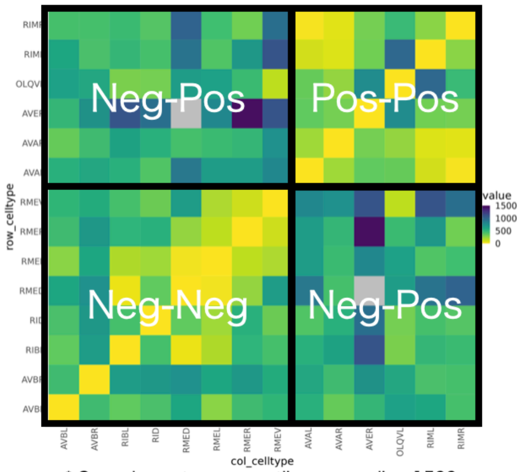

* Gray elements mean outliers exceeding 1500

Figure S14-3 | Time shift values of mSBD between two cells (across all animals). Neg refers to PC1$_{neg}$-related cells, and Pos refers to PC1$_{pos}$-related cells.

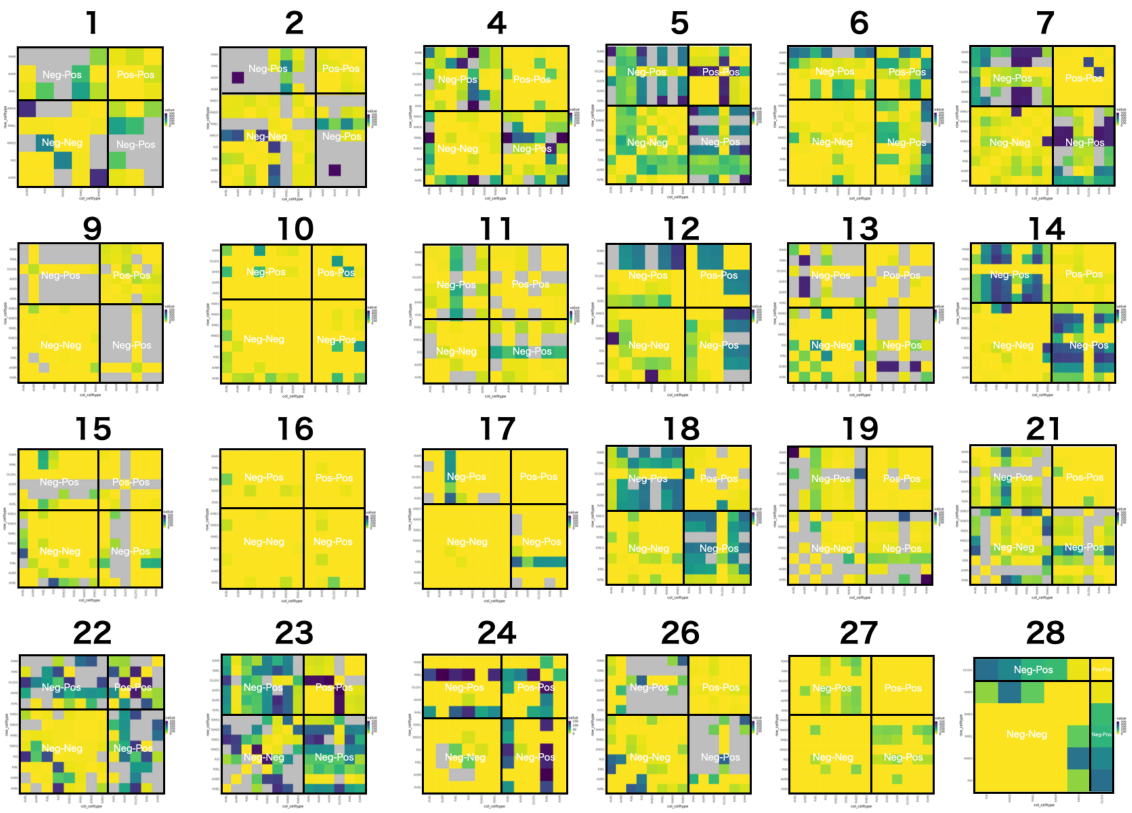

Figure S14-4 | Time shift values of mSBD between two cells (within each animal).
Neg refers to $PC1_{neg}$-related cells, and Pos refers to $PC1_{pos}$-related cells.

Next, we found that the waveforms of $PC1_{pos}$-related cells were not always reciprocal of the waveforms of $PC1_{neg}$-related cells (Figure S14-5). When $PC1_{pos}$-related cells were inactivated, $PC1_{neg}$-related cells were usually, but not always, activated. $PC1_{pos}$- and $PC1_{neg}$-related cells are known to be associated with backward and forward movement, respectively. Although these states are mutually exclusive and reciprocal, sometimes *C. elegans* enters a pause state. This incomplete reciprocity of behavior may correspond to the incomplete reciprocity between waveforms of $PC1_{pos}$- and $PC1_{neg}$-related cells.

From these results, we concluded that $PC1_{pos}$- and $PC1_{neg}$-related cells in our experiment follow some different dynamics and therefore cannot and should not be in the same cluster.

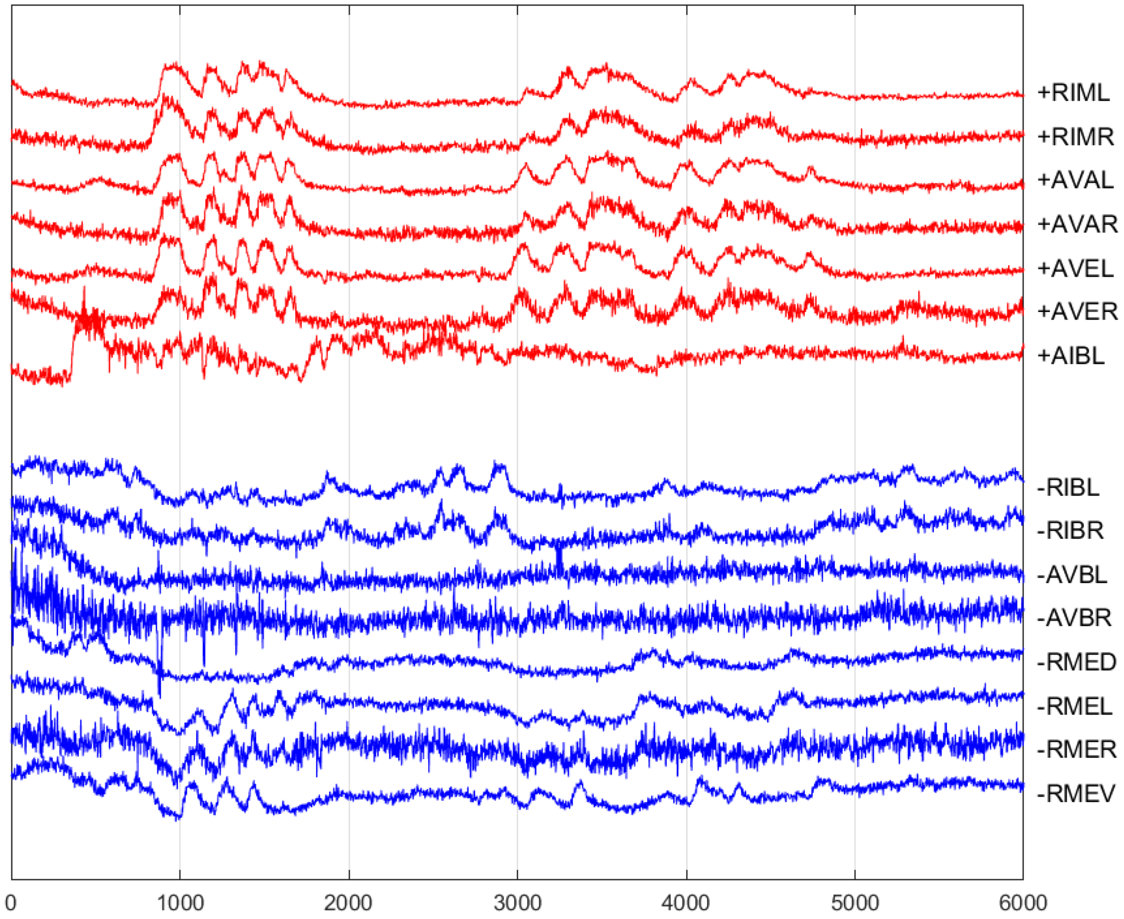

Figure S14-5 | Waveform of time-series neural activity values of PC1$_{pos}$- and PC1$_{neg}$-related cells (based on data from animal 26).

Reference

[1] Kato, S., Kaplan, H.S., Schrodel, T., Skora, S., Lindsay, T.H., Yemini, E., Lockery, S., Zimmer, M.: Global brain dynamics embed the motor command sequence of *Caenorhabditis elegans*. Cell 163(3), 656–669 (2015)
