# Supplementary material for: WormTensor: a clustering method for time-series whole-brain activity data from C. elegans
Source: BMC Bioinformatics. 2023 Jun 16;24:254. doi: 10.1186/s12859-023-05230-2 (PMC10273573; doi:10.1186/s12859-023-05230-2)
Supplement: Supplementary file 15 — Additional file 15. Results for clustering evaluation measures other than silhouette coefficient.PDF 1.68 MB, https://figshare.com/ndownloader/files/38943455. [file 12859_2023_5230_MOESM15_ESM.pdf]

Results for clustering evaluation measures other than silhouette coefficient

In this work, we used the silhouette coefficient [1,2] to evaluate all the clustering results and estimated the optimal number of clusters. Clustering evaluation measures are classified into internal evaluation measures and external evaluation measures [1,2]. Internal evaluation measures including the silhouette coefficient evaluate the clustering result using only the result of clustering itself. Such scores indicate the degree of cluster cohesion and the degree of separation between clusters. Contrary, external evaluation measures evaluate the clustering result using the result of clustering and external measures such as prior knowledge. Such scores indicate the agreement between clusters and external measures.

In our analysis, internal evaluation measures other than the silhouette coefficient could be introduced to estimate the optimal number of clusters. Besides, we also have prior knowledge such as NaCl-related cells and known functional modules as defined by *Saul Kato* et al. [3], we can set such information as external measures and conduct an external evaluation.

However, in our analysis, the measures other than the silhouette coefficient did not successfully estimate the optimal number of clusters. For example, we performed internal measures such as pseudo F-measure, kNN, and connectivity [1,2] (Figure S15-1) but the changes in values were smooth and it was difficult to find the optimal number of clusters as rapidly changing values like the scree plot (elbow plot) in PCA and k-means [1], nor to automatically estimate the number of clusters by choosing the local minimum and maximum.

We also performed ARI, purity, F-measure, and entropy [1,2] as external measures (Figure S15-2) but most of them showed unstable and jagged curves, and the number of clusters was underestimated when the local minimum and maximum values were considered as the optimal clusters (e.g., 2 in ARI). Such a small number of clusters could not separate the functional modules of the cells, which are the subjects of our study. Therefore, such measures were considered inappropriate for our analysis.

Based on the above empirical comparison, we recommend first checking the plot of

silhouette coefficients per cell in WormTensor. All these clustering measures are implemented in the WormTensor package and can be validated by the user on their data.

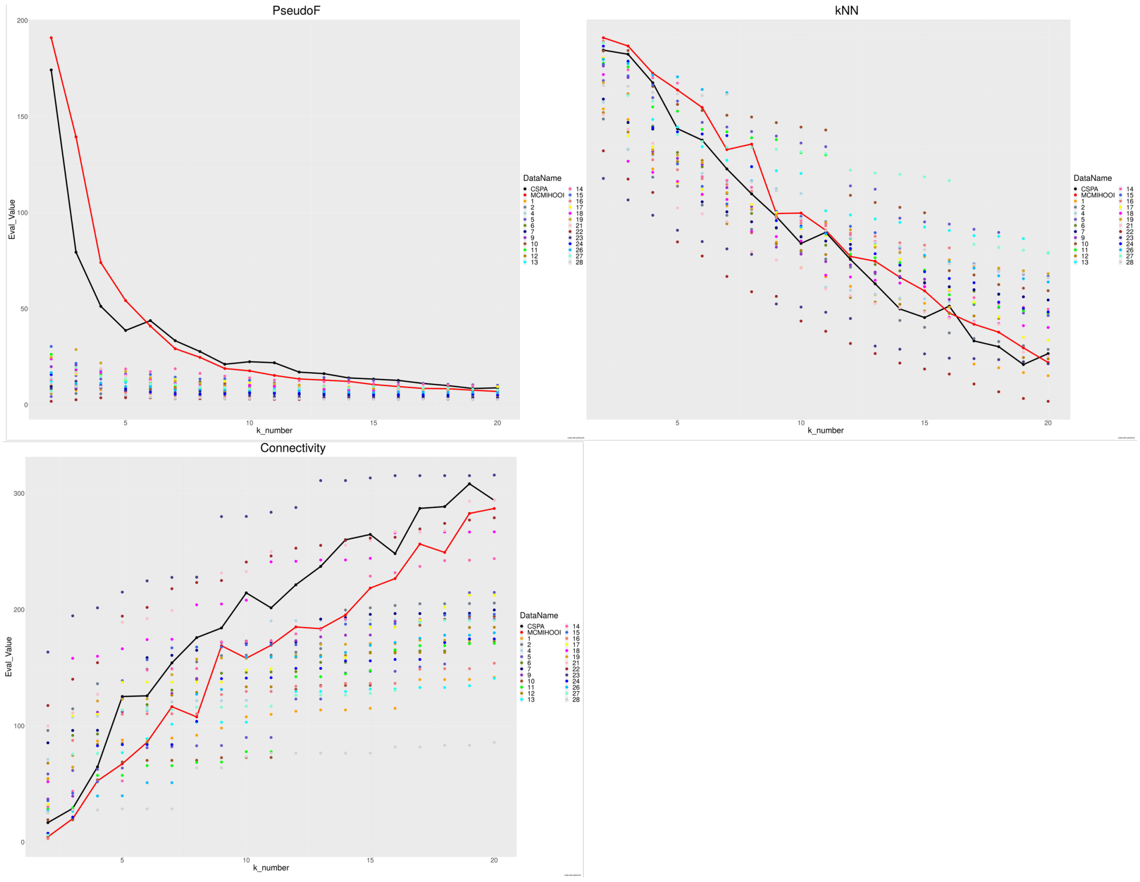

Figure S15-1 | Empirical comparison of internal evaluation for time-series clustering. The x-axis indicates the number of clusters, and the y-axis indicates the value of scores. The black line indicates the results of clustering by CSPA with mSBD. The red line indicates the results of clustering by MC-MI-HOOI with mSBD (WormTensor). The colored dots are the results of hierarchical clustering for each animal. The smaller the connectivity and the larger the other measures, the more cohesive the clusters obtained by clustering and the more separated the clusters are.

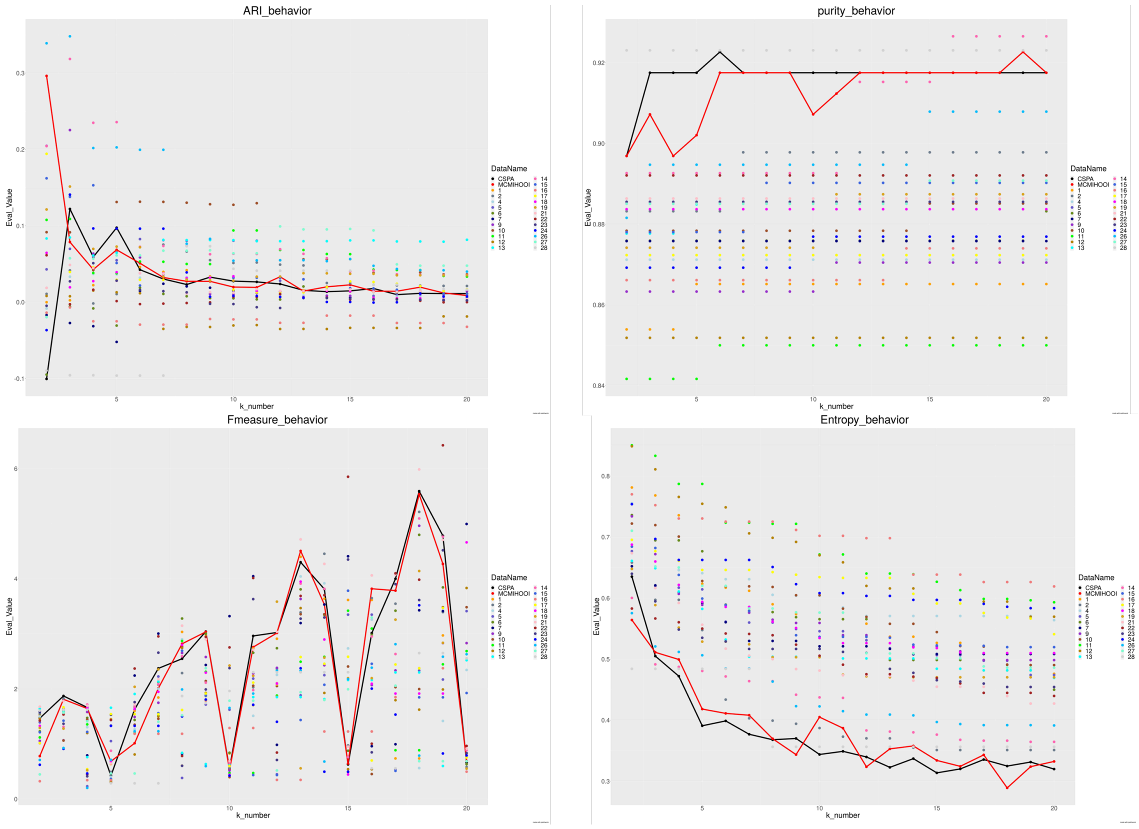

Figure S15-2 | Empirical comparison of external evaluation for time-series clustering. The x-axis indicates the number of clusters, and the y-axis indicates the value of scores. The black line indicates the results of clustering by CSPA with mSBD. The red line indicates the results of clustering by MC-MI-HOOI with mSBD (WormTensor). The colored dots are the results of hierarchical clustering for each animal. The smaller the entropy and the larger the other measures, the more cohesive the clusters obtained by clustering and the more separated the clusters are. The way of separation by clustering is in good agreement with the way of separation by external measures (e.g., prior knowledge).

Reference

[1] Palacio-Niño, J.-O. and Berzal, F. Evaluation Metrics for Unsupervised Learning Algorithms. arXiv, 2019

[2] Tomašev, N. and Radovanović, M. Clustering Evaluation in High-Dimensional Data. Springer, 2016

[3] Kato, S., Kaplan, H.S., Schrodel, T., Skora, S., Lindsay, T.H., Yemini, E., Lockery, S., Zimmer, M.: Global brain dynamics embed the motor command sequence of

Caenorhabditis elegans. Cell 163(3), 656–669, 2015
